# Genetic Drivers of Epigenetic and Transcriptional Variation in Human Immune Cells

**DOI:** 10.1016/j.cell.2016.10.026

**Published:** 2016-11-17

**Authors:** Lu Chen, Bing Ge, Francesco Paolo Casale, Louella Vasquez, Tony Kwan, Diego Garrido-Martín, Stephen Watt, Ying Yan, Kousik Kundu, Simone Ecker, Avik Datta, David Richardson, Frances Burden, Daniel Mead, Alice L. Mann, Jose Maria Fernandez, Sophia Rowlston, Steven P. Wilder, Samantha Farrow, Xiaojian Shao, John J. Lambourne, Adriana Redensek, Cornelis A. Albers, Vyacheslav Amstislavskiy, Sofie Ashford, Kim Berentsen, Lorenzo Bomba, Guillaume Bourque, David Bujold, Stephan Busche, Maxime Caron, Shu-Huang Chen, Warren Cheung, Oliver Delaneau, Emmanouil T. Dermitzakis, Heather Elding, Irina Colgiu, Frederik O. Bagger, Paul Flicek, Ehsan Habibi, Valentina Iotchkova, Eva Janssen-Megens, Bowon Kim, Hans Lehrach, Ernesto Lowy, Amit Mandoli, Filomena Matarese, Matthew T. Maurano, John A. Morris, Vera Pancaldi, Farzin Pourfarzad, Karola Rehnstrom, Augusto Rendon, Thomas Risch, Nilofar Sharifi, Marie-Michelle Simon, Marc Sultan, Alfonso Valencia, Klaudia Walter, Shuang-Yin Wang, Mattia Frontini, Stylianos E. Antonarakis, Laura Clarke, Marie-Laure Yaspo, Stephan Beck, Roderic Guigo, Daniel Rico, Joost H.A. Martens, Willem H. Ouwehand, Taco W. Kuijpers, Dirk S. Paul, Hendrik G. Stunnenberg, Oliver Stegle, Kate Downes, Tomi Pastinen, Nicole Soranzo

**Affiliations:** 1 Department of Human Genetics, The Wellcome Trust Sanger Institute, Wellcome Trust Genome Campus, Hinxton, Cambridge CB10 1HH, UK; 2 Department of Haematology, University of Cambridge, Cambridge Biomedical Campus, Long Road, Cambridge CB2 0PT, UK; 3 Human Genetics, McGill University, 740 Dr. Penfield, Montreal, QC H3A 0G1, Canada; 4 European Molecular Biology Laboratory, European Bioinformatics Institute, Wellcome Genome Campus, Hinxton, Cambridge CB10 1SD, UK; 5 Bioinformatics and Genomics, Centre for Genomic Regulation (CRG), Barcelona Institute of Science and Technology, Carrer del Dr. Aiguader, 88, Barcelona 8003, Spain; 6 Department of Experimental and Health Sciences, Universitat Pompeu Fabra (UPF), Plaça de la Mercè, 10- 12, Barcelona 8002, Spain; 7 Structural Biology and Biocomputing Programme, Spanish National Cancer Research Centre (CNIO), Melchor Fernandez Almagro, 3, Madrid 28029, Spain; 8 UCL Cancer Institute, University College London, 72 Huntley Street, London WC1E 6BT, UK; 9 Vertebrate Genomics, European Molecular Biology Laboratory, European Bioinformatics Institute, Wellcome Trust Genome Campus, Hinxton, Cambridge CB10 1SD, UK; 10 Genome Analysis, European Molecular Biology Laboratory, European Bioinformatics Institute, Wellcome Trust Genome Campus, Hinxton, Cambridge CB10 1SD, UK; 11 European Molecular Biology Laboratory, European Bioinformatics Institute, Wellcome Trust Genome Campus, Hinxton, Cambridge CB10 1SD, UK; 12 Genetic Medicine and Development, University of Geneva Medical School-CMU, 1 Rue Michel-Servet, Geneva 1211, Switzerland; 13 Human Genetics, Donders Institute for Brain, Cognition and Behaviour, Radboud University Medical Centre, P.O. Box 9101, Nijmegen 6500 HB, the Netherlands; 14 Department of Vertebrate Genomics, Max Planck Institute for Molecular Genetics, Ihnestr. 63/73, Berlin 14195, Germany; 15 Department of Molecular Biology, Faculty of Science, Radboud University, Nijmegen 6525GA, the Netherlands; 16 Molecular Developmental Biology, Radboud Institute for Life Sciences, Radboud University, P.O. Box 9101, Nijmegen 6500 HB, the Netherlands; 17 Human Genetics Informatics, The Wellcome Trust Sanger Institute, Wellcome Trust Genome Campus, Hinxton, Cambridge CB10 1HH, UK; 18 National Health Service (NHS) Blood and Transplant, Cambridge Biomedical Campus, Long Road, Cambridge CB2 0PT, UK; 19 Institute for Systems Genetics, New York University Langone Medical Center, ACLS West, Room 511, 430 East 29^th^ Street, New York, NY 10016, USA; 20 Blood Cell Research, Sanquin Research and Landsteiner Laboratory, Plesmanlaan 125, Amsterdam 1066CX, the Netherlands; 21 Bioinformatics, Genomics England, Charterhouse Square, London EC1M 6BQ, UK; 22 British Heart Foundation Centre of Excellence, Division of Cardiovascular Medicine, Addenbrooke’s Hospital, Hills Road, Cambridge CB2 0QQ, UK; 23 Computational Genomics, Institut Hospital del Mar d’Investigacions Mediques (IMIM), Carrer del Dr. Aiguader, 88, Barcelona 8003, Spain; 24 Institute of Cellular Medicine, Newcastle University, Framlington Place, Newcastle upon Tyne NE2 4HH, UK; 25 The National Institute for Health Research Blood and Transplant Unit (NIHR BTRU) in Donor Health and Genomics at the University of Cambridge, Strangeways Research Laboratory, University of Cambridge, Wort’s Causeway, Cambridge CB1 8RN, UK; 26 Emma Children’s Hospital, Academic Medical Center (AMC), University of Amsterdam, Location H7-230, Meibergdreef 9, Amsterdam 1105AZ, the Netherlands; 27 Cardiovascular Epidemiology Unit, Department of Public Health and Primary Care, Strangeways Research Laboratory, University of Cambridge, Wort’s Causeway, Cambridge CB1 8RN, UK

**Keywords:** immune, monocyte, neutrophil, t-cell, EWAS, histone modification, DNA methylation, transription, allele specific, QTL

## Abstract

Characterizing the multifaceted contribution of genetic and epigenetic factors to disease phenotypes is a major challenge in human genetics and medicine. We carried out high-resolution genetic, epigenetic, and transcriptomic profiling in three major human immune cell types (CD14^+^ monocytes, CD16^+^ neutrophils, and naive CD4^+^ T cells) from up to 197 individuals. We assess, quantitatively, the relative contribution of *cis*-genetic and epigenetic factors to transcription and evaluate their impact as potential sources of confounding in epigenome-wide association studies. Further, we characterize highly coordinated genetic effects on gene expression, methylation, and histone variation through quantitative trait locus (QTL) mapping and allele-specific (AS) analyses. Finally, we demonstrate colocalization of molecular trait QTLs at 345 unique immune disease loci. This expansive, high-resolution atlas of multi-omics changes yields insights into cell-type-specific correlation between diverse genomic inputs, more generalizable correlations between these inputs, and defines molecular events that may underpin complex disease risk.

## Highlights

• Genome, transcriptome, and epigenome reference panel in three human immune cell types
• Identified 4,418 genes associated with epigenetic changes independent of genetics
• Described genome-epigenome coordination defining cell-type-specific regulatory events
• Functionally mapped disease mechanisms at 345 unique autoimmune disease loci

## Graphical Abstract

**Figure:**
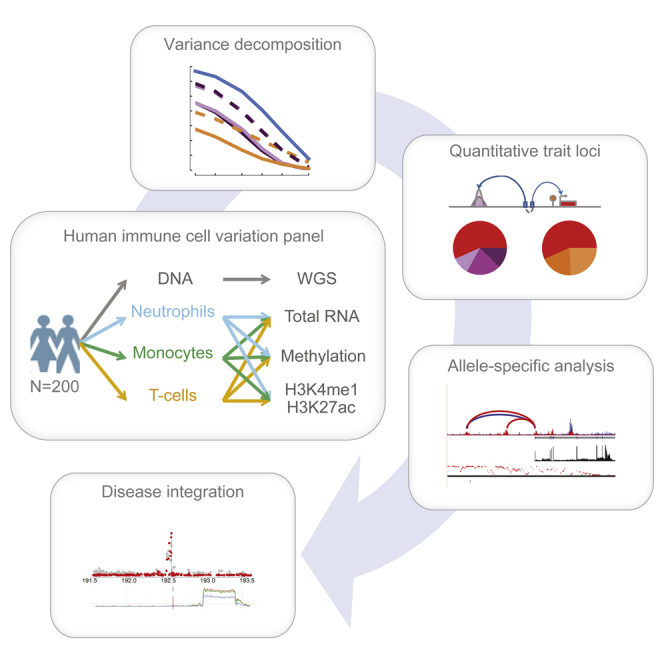

## Teaser

As part of the IHEC consortium, this study integrates genetic, epigenetic, and transcriptomic profiling in three immune cell types from nearly 200 people to characterize the distinct and cooperative contributions of diverse genomic inputs to transcriptional variation. Explore the Cell Press IHEC web portal at http://www.cell.com/consortium/IHEC.

## Introduction

Many human complex diseases are characterized by dysregulation of immune and inflammatory activity. However, the repertoire of immune genes and cell subsets implicated in the pathogenesis of individual disease can vary dramatically. Genome-wide association studies (GWAS) have contributed to expanding catalogs of implicated genes and pathways for many complex human diseases (Hindorff et al., 2009) and are beginning to shed light on shared and unique etiological and pathological components of disease (Farh et al., 2015, Jostins et al., 2012). A key challenge is that these disease variants map predominantly to noncoding regions of the human genome, where they are predicted to alter regulatory function (Kundaje et al., 2015). Linking susceptibility variants to their respective causative genes and cell-specific regulatory elements thus remains a main priority in order to realize the potential of association studies to advance understanding of disease biology and etiology, leading to therapeutic advances.

Molecular quantitative trait locus (QTL) studies testing for associations between genetic variants and intermediate phenotypes, in particular gene expression levels, provide powerful approaches to annotate the putative consequence of disease associations (Montgomery and Dermitzakis, 2011). The biological resolution of this approach can be further increased using two main strategies. First, genetic effects on gene expression have been shown to be often context-specific (Kundaje et al., 2015) and thus are better captured in studies probing multiple primary cell types or experimental conditions (Bentham et al., 2015, Fairfax et al., 2014, Naranbhai et al., 2015). Second, extending these analyses beyond gene expression to other molecular phenotypes such as variable histone modification or methylation status can greatly enhance the functional and mechanistic interpretation of genetic associations (Allum et al., 2015). Recent studies in cell line models have demonstrated the occurrence of a high degree of local coordination between transcriptional and epigenetic states and suggested that a fraction of disease-associated genetic variants may alter expression levels through changes in chromatin state (Grubert et al., 2015, Waszak et al., 2015). Extending these integrated investigations to primary human cells in disease-relevant contexts is the necessary next step to unravel the cell- and context-specific regulatory effects of complex disease variants.

Here, we report an integrated analysis of genetic, epigenetic, and transcriptomic datasets in the three major cells of the human immune system, namely CD14^+^ monocytes, CD16^+^ neutrophils, and CD4^+^ naive T cells. Monocytes contribute to maintenance of the resident macrophage pool under steady-state conditions and migrate to sites of infection in the tissues and divide/differentiate into macrophages and dendritic cells to elicit an immune response. Neutrophil granulocytes (neutrophils) are primary blood cells of the innate immune and inflammatory response system that form a first line of organismal response to bacterial and fungal infection, migrating within minutes to sites of infection, attracted by local tissue factors and resident macrophages during the acute phase of inflammation. Finally, CD4^+^ naive T cells are part of the adaptive immune response system, representing mature helper T cells that have not yet encountered their cognate antigen.

We generated high-resolution whole-genome sequence, transcriptome, DNA methylation, and histone modification datasets in up to 197 individuals selected from a population-based sample and applied variance decomposition, QTL, and allelic imbalance analyses to investigate genetic and epigenetic influences to transcription and RNA splicing in the three primary immune cells. We demonstrate colocalization of molecular trait QTLs with 345 unique genetic variants predisposing to seven human autoimmune diseases, involving all data layers. Overall, the data and results deepen our understanding of genetic and epigenetic regulation of the transcriptional machinery in three primary cells of the immune system and inform the formulation and testing of functional hypotheses for human complex disease.

## Results

### Study Design

**Figure 1:**
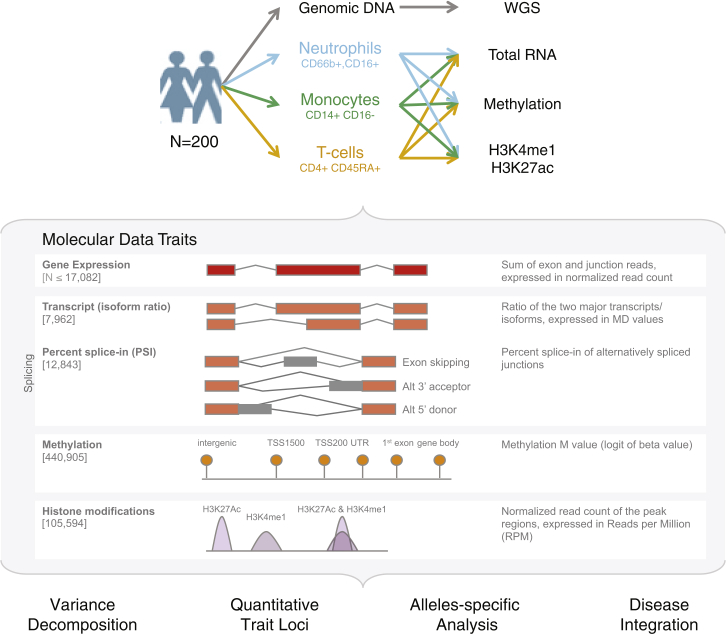
Study Design Overview of study design and molecular traits investigated. Details of sample collections are given in Figure S1 and Table S1.

**Figure S1:**
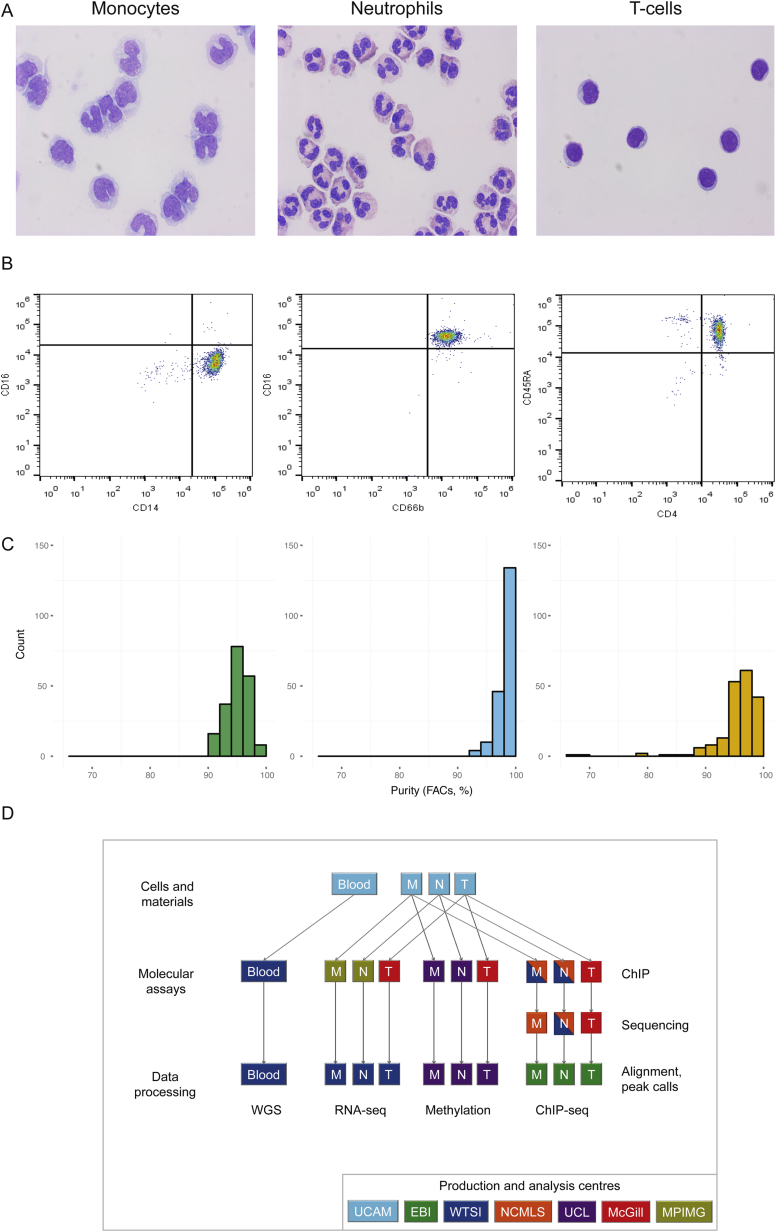
Sample Collection, Related to Figure 1 (A) Morphological assessment of purified cell preparations. Cells were fixed to slides using a Cytospin and stained using Wright-Giemsa stain prior to photographing using 100x magnification. (B) Examples of neutrophil, monocyte and naive CD4+ T cell staining to assess purity of cell preparations. (C) Histogram of cell purity based on FACs analysis in three cell types. (D) Details of data production centers. Data from this project were produced in different institutes as detailed here: University of Cambridge- UCAM, European Bioinformatics Institute- EBI, Wellcome Trust Sanger Institute- WTSI, Nijmegen Centre for Molecular Life Sciences- NCMLS, University College London- UCL, McGill University- McGill, Max Planck Institute for Molecular Genetics- MPIMG. Peripheral blood mononuclear cells (PBMC) were isolated from donors at UCAM and from these Monocytes (M), Neutrophils (N), naive CD4+ T cells (T) were extracted, with a further aliquot used as a source of genomic (g)DNA samples. gDNA was shipped to the WTSI for sequencing, the monocyte/neutrophil samples were divided between MPIMG/UCL/WTSI+NCMLS for RNA-seq, DNA methylation sequencing (Methylation) and ChIP-seq respectively and the naive CD4+ T cells sent to McGill for RNA-seq/Methylation/ChIP-seq. In addition to this three samples from each institute/assay set were sent to the reciprocal institute for cross-center validation purposes (eg RNA-seq assays were carried out on the same three samples at both MPIMG and McGill etc.). Data processing/analysis was carried out at WTSI for WGS and RNA-seq, UCL for DNA methylation sequencing and EBI for ChIP-seq.

As part of the BLUEPRINT epigenome project, we recruited an initial set of 200 blood donors from a local blood donor population, ascertained to be free of disease and representative of the United Kingdom (UK) population at large (54% females, mean age 55 years) (Figure 1; Table S1). We used a multi-step purification strategy (Figure S1) to isolate, for each donor, cell subsets corresponding to classical monocytes (CD14^+^CD16^−^) and neutrophils (CD66b^+^CD16^+^). Subsequently, through a collaboration with Epigenome Mapping Centre at McGill University, we were able to extend the study to a third cell type (“phenotypically naive” CD4^+^CD45RA^+^ T cells, henceforth referred to as CD4^+^ T cells or T cells for simplicity) for 169 out of the 200 donors.

**Figure S2:**
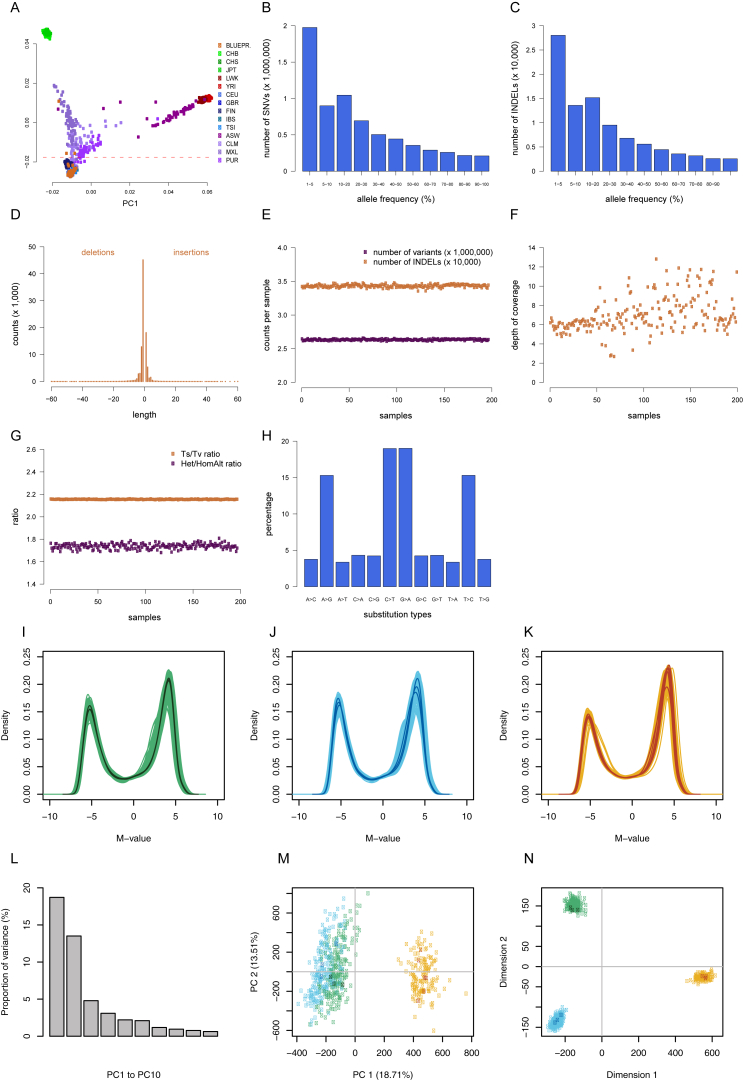
WGS and DNA Methylation Sample and Data Quality Metrics, Related to Figures 3 and 4 WGS (A-H) and DNA methylation (I-N) sample and data quality metrics. (A) Principal component analysis (PCA) scatterplot of the first two components using the resulting merged datasets (1000GP + Blueprint). The dashed line indicates the arbitrary threshold to discriminate the population of European ancestry. (B) Number of SNPs (x10^6^) by non-reference allele frequency (AF) bins. (C) Number of INDELs (x10^4^) by non-reference AF bins. (D) Size distribution of INDELs. Negative lengths represent deletions and positive lengths represent insertions. (E) Number of SNPs (x10^6^) and INDELs (x10^4^) by sample. (F) Depth of coverage by sample. (G) Ratio of heterozygous and homozygous non-reference SNP genotypes by sample and transition to transversion ratio (Ts/Tv) by sample. (H) Types of substitution in percentage. (I-K) Distributions of DNA methylation M-values for each cell type. Each line represents one sample. (L) Barplot representing the proportions of variance explained by the first ten principal components of a principal component analysis across all samples used in the study. (M) Visualization of the first two principal components of a principal component analysis across all samples used in the study. Each data point represents one sample, colored by cell type. (N) Multidimensional scaling of all samples used in the study, based on Euclidean distances. Each data point represents one sample, colored by cell type.

**Figure S3:**
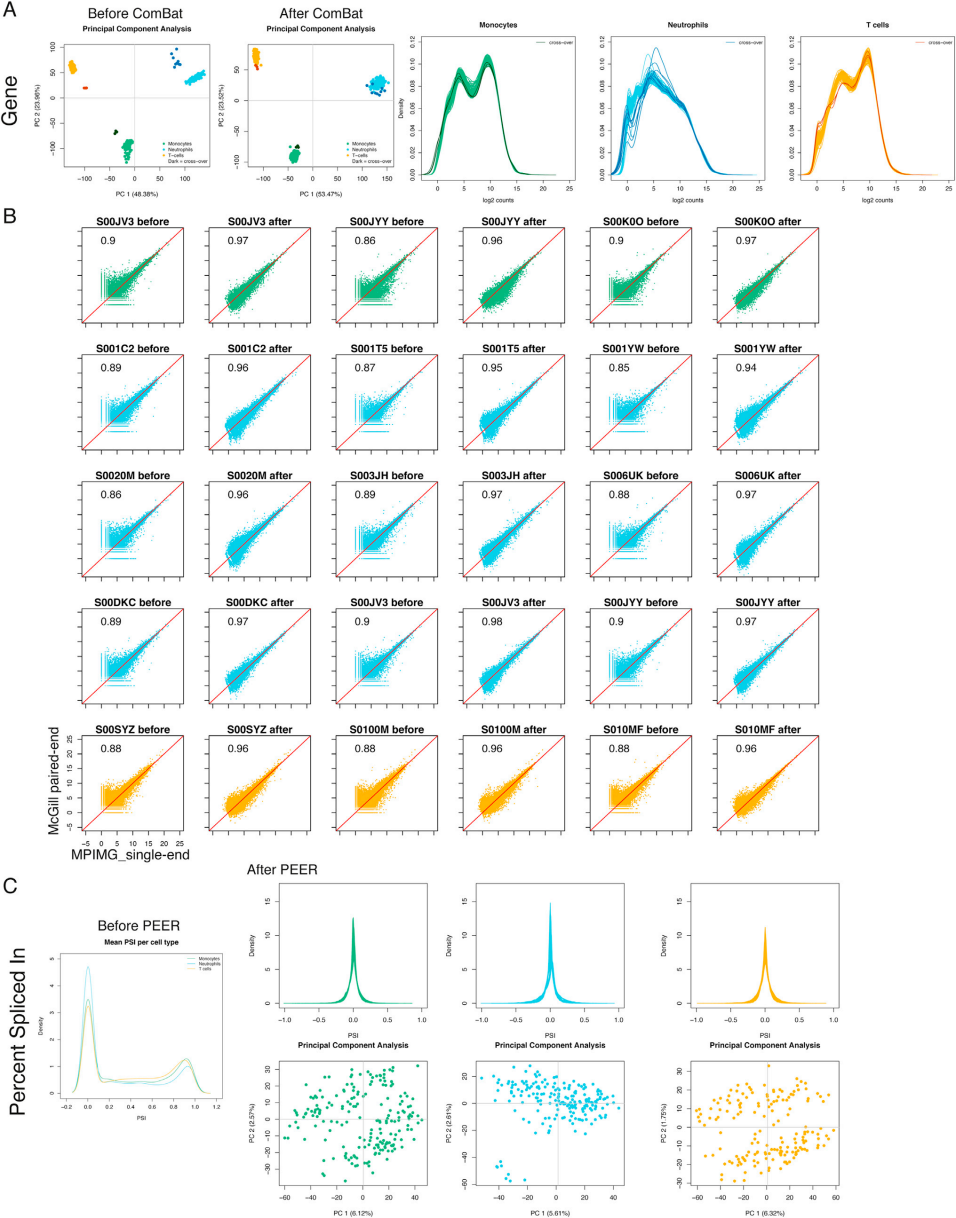
RNA-Seq Distribution and Batch Correction, Related to Figures 3 and 4 (A) PCA before and after batch correction using ComBat in gene level. Darker color lines and dots represent cross-over samples from different sequence center. Distribution of normalized read counts (log2) in gene level in monocytes, neutrophils and naive CD4+ T cells. (B) Scatterplots of the pairwise correlation of gene quantification between crossover samples before and after batch correction. (C) Distribution of before and after PEER corrected PSI values (upper panel) and PCA plots (lower panel) in monocytes, neutrophils and naive CD4+ T cell.

**Figure S4:**
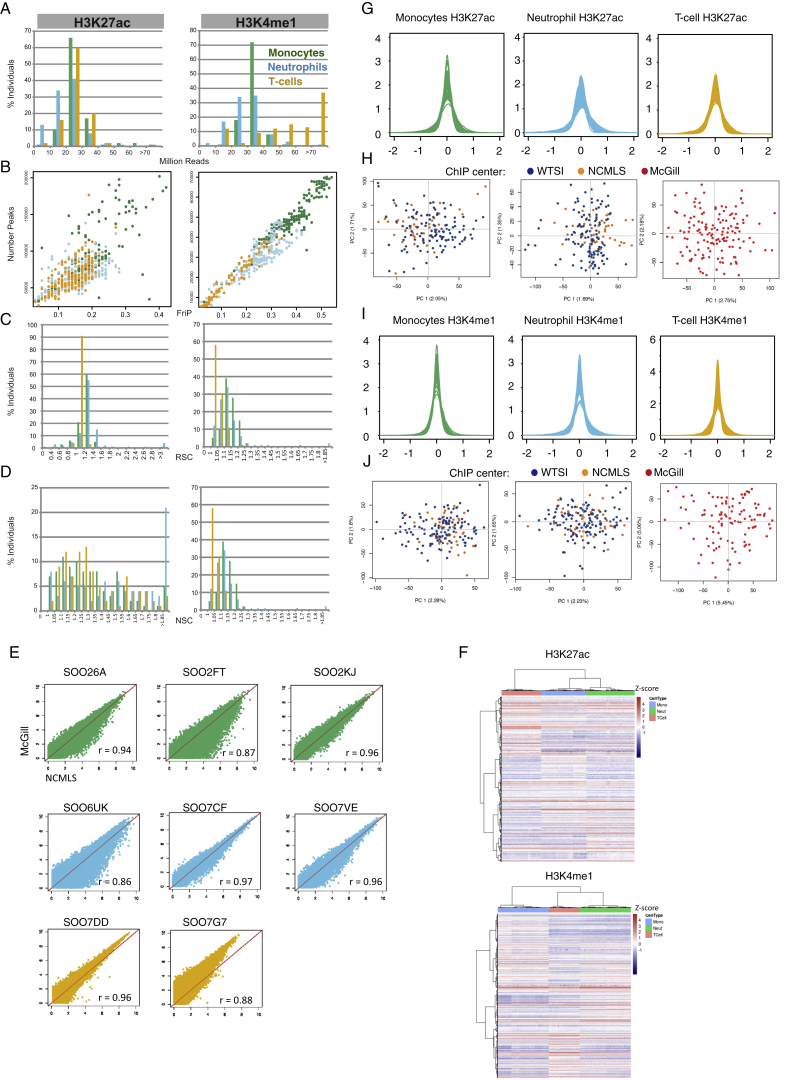
ChiP-Sequencing Data Quality Metrics, Related to Figure 3 (A–D) ChIP-seq quality control plots with consistent color convention throughout; Neutrophil (blue), Monocyte (green) and T cell (yellow). Plots are split by factor assayed, H3K4me1 (left) and H3K27ac (right). (A) Histogram displaying bins of quality control passed reads on x axis and percent of individuals falling into each bin on y axis. (B) Scatterplot displaying number of peaks called at FDR threshold per individual and colored by cell type is shown on x axis. On the y axis fraction of reads intersecting a consensus peak set of regions shared across all three cell types. (C) Histogram displaying bins of normalized strand coefficient on x axis, y axis percent of individual which fall into each bin. (D) Histogram displaying bins of relative strand coefficient on x axis, y axis percent of individual which fall into each bin. (E and F) Scatterplot showing the Pearson correlation r between replicates of same donors processed at NCMLS and McGill (F) Hierarchical clustering for each histone modification marker using Pearson correlation as distance metrics and standardized log2 RPM (Reads Per Million) in chromosome 1 only. Similar clustering is likewise seen for all chromosomes. (G–J) PEER corrected matrices of log2 RPM. Density of log2 RPM values for H3K27ac in (G) and for H3Kme1 in (I). Scatterplot colored by the ChIP center of the first two orthogonal components from PCA for H3K27ac in (H) and for H3Kme1 in (J).

For each individual, we performed whole-genome sequencing (WGS) (mean read depth, approximately 7×) (Figure S2; Table S1) and probed the transcriptional profiles (RNA sequencing [RNA-seq] at ∼80 million reads per sample) (Figure S3), genome-wide DNA methylation (Illumina 450K arrays) (Figure S2), and two histone modification marks for active and poised enhancers and active promoters (H3K4me1 and H3K27ac, chromatin immunoprecipitation sequencing [ChiP-seq] at ≥30 million reads per sample) (Figure S4). Molecular assays for monocytes and neutrophils were distributed across four laboratories, and assays on T cells were done at McGill (Figure S1). We carefully assessed and adjusted for possible sequencing artifacts that may arise due to differences in protocols between centers, applying stringent quality filters where needed. We confirmed that our approach avoided significant effects by profiling a subset of the same individuals across each respective center (“cross-over experiments”) (Figures S1, S2, S3, and S4; Table S2). Overall, the project generated 116,310 million QC-pass reads across all datasets, with 80% of donors passing ten or more assays and 56 donors having complete data across all cell types and molecular assays (Table S1).

### Decomposition of Transcriptional Variance into Genetic and Epigenetic Components

Matched genetic, epigenetic, and gene expression profiles from multiple donors in this study provides a unique opportunity to characterize the relationship between hierarchies of gene regulation and how these regulatory links ultimately affect human phenotypic variation. Detailed understanding of this relationship is necessary for the correct interpretation of the contribution of epigenetic variation to organismal traits and disease.

**Figure 2:**
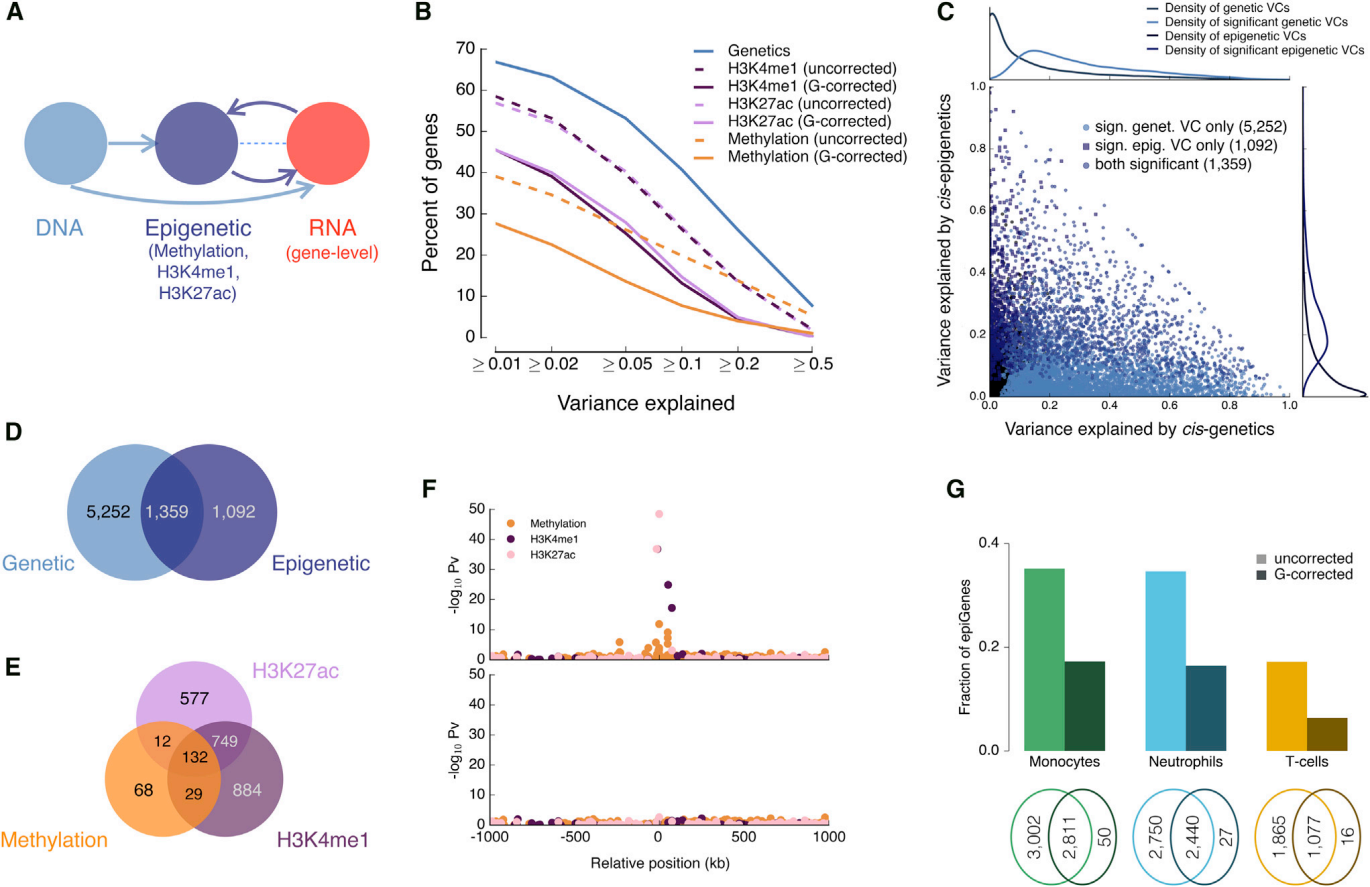
Variance Decomposition and Epigenetic Association Analysis of Gene Expression (A) Mechanisms of genetic and epigenetic associations with gene expression. Considered are direct *cis*-acting genetic effects (light blue) as well as epigenetic correlations with gene expression that are independent of genetics (dark blue). No assumption is made on causal directionality for shared genetic effects (light blue, dashed line). (B) Proportion of transcriptome variance explained by genetic and epigenetic factors for individual genes, when considering putative *cis*-regulatory elements (within ±1 Mb of the gene body). Shown is the cumulative contribution of genes with increasing proportions of explained variance, considering genetic factors (blue), DNA methylation (orange), H3K4me1 (violet), and H3K27ac (pink) in monocytes. Epigenetic variance components were estimated either with (solid lines, G-corrected) or without (dashed lines, uncorrected) accounting for local *cis*-genetic variation (Methods). (C) Scatterplot of the proportion of variance explained by *cis*-genetics (x axis) versus *cis*-epigenetic (y axis) effects in monocytes. Significant variance components (VCs, FDR <5%) are coded in color. (D) Overlap of genes with significant *cis*-genetic and *cis*-epigenetic contributions to expression variance. (E) Overlap of genes with significant contributions from (*cis*) DNA methylation, (*cis*) H3K4me1, and (*cis*) H3K27ac. (F) Manhattan plot for gene *TMEM176A* obtained from the *cis*-epigenetic association analysis of gene expression in monocytes. Top panel: analysis without accounting for *cis*-genetic variation. Bottom panel: analysis when accounting for *cis*-genetic variation. (G) Fraction of genes with significant epigenetic associations (epiGenes, FDR <5%) before (uncorrected) and after correcting (G-corrected) for common *cis*-genetic variation. For T cells, a lower number of ChIP-seq data for H3K4me1 was due to lower initial immunoprecipitation enrichment for a subset of cryopreserved samples with insufficient material for repeat assays; hence only methylation was used in this analysis. See also Figures S5 and S6 and Tables S3 and S4.

**Figure S5:**
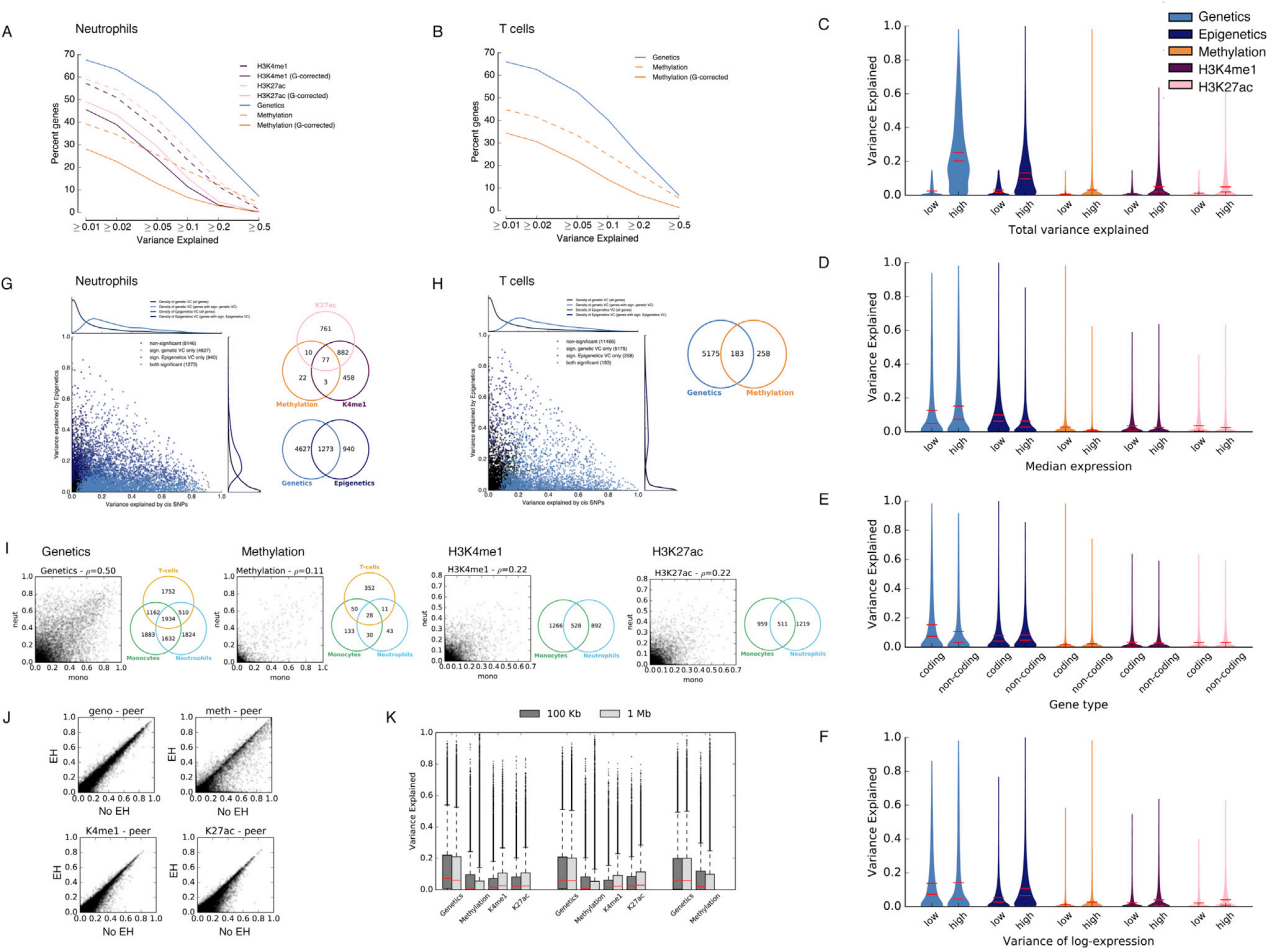
Variance Decomposition Analyses, Related to Figure 2 (A and B) Figures showing analogous results as those presented in Figure 2B; however, for neutrophils and naive CD4+ T cells. (C–F) Variance partitioning results obtained from the joint model across all four molecular layers in monocytes. Shown are the distributions of variance explained by genetics, cumulative epigenetics as well as separately for individual epigenetic layers for different sets of genes. Specifically, genes were stratified by the median of (C) the total variance explained by the joint model (“low” and “high” indicate genes below and above the median), (D) the median gene-expression level, (E) gene type and (F) the variance of the log of the expression levels. (G and H) Figures showing analogous results as those presented in Figures 2C–2E; however for neutrophils and naive CD4+ T cells. (I) Pairwise correlation of the variance explained by different molecular layers between monocytes and neutrophils. Epigenetic contributions were estimated using a model that accounts for underlying genetic variation (see the STAR Methods). The Spearman’s rank correlation (ρ) is also reported. Venn Diagrams show the overlap of genes with significant genetic, methylation, H3K4me1 and H3K27ac contributions between monocytes and neutrophils (FDR < 5%, using a variance component test, see the STAR Methods). (J) Comparison of variance component estimates for individual molecular layers either considering a model that accounts for expression heterogeneity (EH, y axis) or a model that does not account for EH (no EH, x axis) in monocytes (see the STAR Methods). The genetic variance estimates were consistent across both approaches, whereas epigenetic variance estimates were substantially increased when not using the additional EH adjustment. (K) Comparison of the proportion of variance explained by different molecular layers across cell types when either considering a 100Kb or a 1Mb *cis* window (see the STAR Methods).

We first sought to quantify the relative impact of genetic and epigenetic factors to transcriptional variance. Associations between epigenetic and RNA traits may arise from two potential causes: (1) local epigenetic changes that correlate with RNA level but themselves are due to DNA sequence variation (Figure 2A), and (2) epigenetic changes that are correlated with RNA level and not associated with *cis* genetic variation. To quantify the relative contribution of genetic and epigenetic factors to transcriptional variance, we fit variance decomposition models (Lippert et al., 2014, Casale et al., 2015) to explain transcriptome variance using common genetic (minor allele frequency [MAF] >4%) and epigenetic features within 1 Mb of the gene. DNA methylation and histone modifications explained lower proportion of transcriptome variance in models where epigenetic elements were adjusted for proximal genetic effects compared to the corresponding unadjusted models in all cell types (Figures 2B, S5A, and S5B), suggesting that genetic effects are the main determinant of transcriptome variance.

We next fit a joint model that considers all four molecular layers (genetic, methylation, H3K4me1, and H3K27ac). Globally, the proportion of expression variance explained by epigenetic effects (average 3.2% for H3K4me1, 3.1% for H3K27ac, and 1.9% for methylation in monocytes) was small compared to genetic effects (average 13.9% in monocytes) (Figures S5C–S5F). Estimates of the overall contribution of DNA methylation is conservative in this analysis, because methylation sites are incompletely ascertained in the Illumina 450k array (representing ∼2% of all annotated CpGs, for 99% of RefSeq genes at mainly promoters and genic enhancers). When testing for significance of the variance components in this model, we identified 2,451, 2,213, and 441 genes with significant epigenetic component (false discovery rate [FDR] <5%) in monocytes, neutrophils, and T cells, respectively, of which 1,092, 940, and 258 genes had no significant genetic effect (Figures 2C–2E, S5G, and S5H; FDR <5%). These results indicate that some local epigenetic associations with RNA cannot be explained by shared genetic effects due to common variants. These genes were implicated in key functions in innate and acquired immunity and inflammation. As examples, genes of the inflammasome pathway were strongly enriched in neutrophils (p = 2 × 10^−6^; Table S3). Inflammasomes are innate immune system complexes that regulate the activation of caspase-1 and the proinflammatory *IL-1* family of cytokines. This process is induced by detection of pathogen-associated molecular patterns (PAMPs) or danger-associated molecular patterns (DAMPs) culminating in the induction of inflammation in response to infectious microbes and molecules derived from host damage. Inflammasomes have also been implicated in a range of inflammatory processes and disorders. In monocytes, we detected epigenetic influences for genes within a number of key signaling pathways involved in the immune cell function, including the Tec kinase and eicosanoid signaling pathway, the nuclear factor κB (*NF-κB*), *CXCL8*, and interleukin-10 (*IL-10*) signaling pathways (Melcher et al., 2008, Schmidt et al., 2004) (Table S3). These findings suggest that function related to pathogen response may be primed and controlled at least in part through epigenetic rather than genetic mechanisms. Finally, estimated variance components of individual genes were correlated across cell types, most significantly for genetic factors, but also for pure epigenetic variance contributions (Figure S5I).

**Figure S6:**
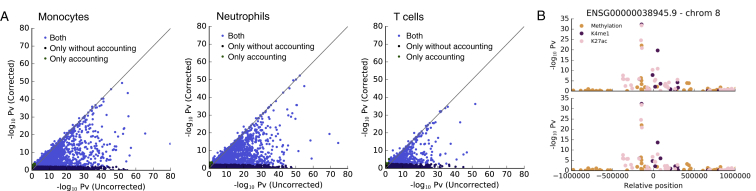
EWAS, Related to Figure 2 (A) Scatterplot of the gene-level P values (see the STAR Methods) obtained from the EWAS analysis either accounting (y axis) or not (x axis) for genetic effects in all three cell types. Genes with significant *cis*-epigenetic association only when not accounting for underlying genetic effects (“Only without accounting,” FDR < 5%) are indicated in dark blue. Genes with significant *cis*-epigenetic association only when accounting for underlying genetic effects (“Only accounting”) are indicated in green. Finally, genes with significant *cis*-epigenetic associations both when accounting or not for underlying genetic effects (“Both”) are indicated in blue. (B) Manhattan plot for the gene *MSR1* (ENSG00000038945), illustrating a *cis* epigenetic association that is robust to correction of genetic effects. Shown are -log10(pv) from an EWAS analysis either without accounting for *cis* genetic effects (top panel) or when accounting for *cis* genetic variation (bottom panel).

The large prevalence of epigenome-transcriptome associations that can be attributed to shared genetic effects may have important implications for interpretation of epigenome-wide association studies (EWAS). To explore this possibility, we next considered gene expression measured from RNA sequencing experiments as a proxy for organismal and disease phenotype. We then carried out a conventional EWAS analysis testing for association between epigenetic traits (within 1 Mb of the gene) and gene expression without accounting for *cis*-genetics and compared it to a second model where we adjusted for local *cis*-genetic effects (variants within 1 MB of the gene body). In the traditional EWAS, we identified significant epigenome associations with gene expression for between 35% (5,813 in monocytes and 5,190 genes in neutrophils, FDR <5%) (Figures 2F and 2G) and 16.5% of the genes tested (2,942 genes in T cells, where the model was fitted only on methylation owing to the smaller H3K4me1 dataset). However, when accounting for *cis*-genetic effects, >50% of the genes with an EWAS signal were no longer significant (Figures 2F, 2G, and S6A). This demonstrates that failure to account for genetic factors in EWAS may lead to an overestimation of the total contribution of epigenetic factors to phenotype. The magnitude of this effect may also vary in disease-focused studies.

Overall, our results demonstrate that a large part of epigenetic associations with transcriptome variance at population level are correlated with underlying common *cis*-genetic variation, consistent with a high degree of local coordination between genetic, epigenetic, and transcriptional variation (Grubert et al., 2015, Waszak et al., 2015). We further show that this correlation is an important confounder in epigenome-wide association studies. Notably, however, careful integrative statistical modeling can identify clear epigenetic influences independent of *cis*-genetic factors for classes of biologically relevant genes.

### Coordinated Influence of QTL Variants across Molecular Data Layers

**Figure 3:**
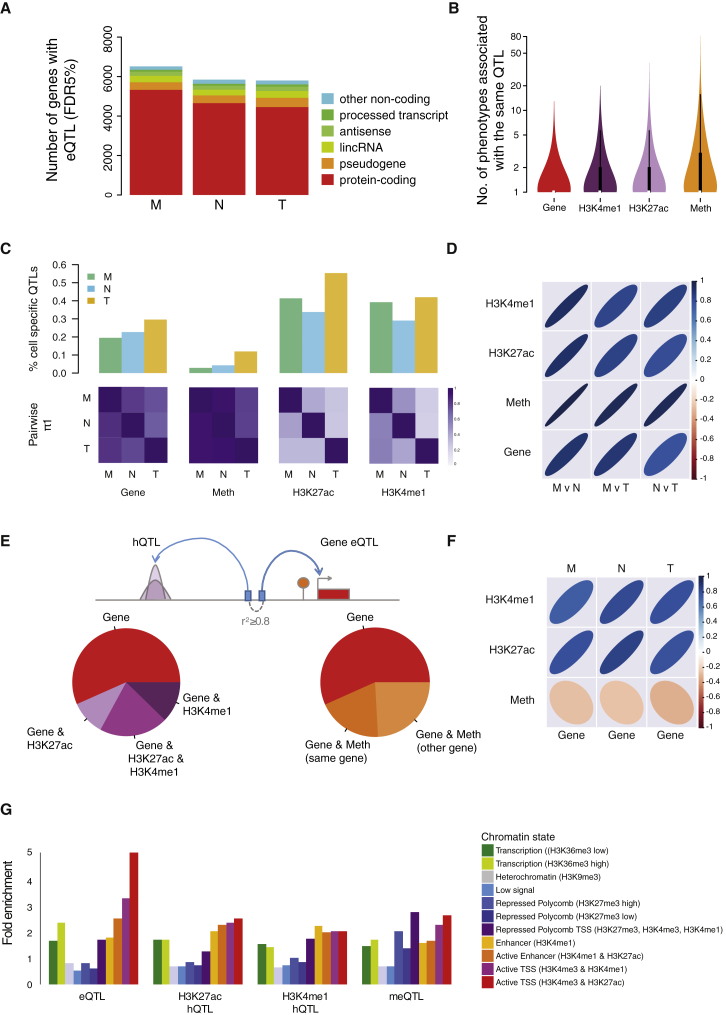
Features, Cell-type Specificity, and Coordination of QTLs (A) Number of protein-coding and non-coding genes with significant eQTL (FDR <5%). (B) Number of phenotypes associated with the same QTL. (C) Percentage of phenotypes that are cell-type-specific (top) and genome-wide patterns of QTL sharing (π1 statistics) among cell types (bottom). (D) Correlation (Pearson) between effect sizes for QTLs shared between different cell types. (E) Percentage of eSNPs also associated (r^2^ ≥ 0.8) with H3K27ac and H3K4me1 (left) or methylation levels (right). (F) Correlation (Pearson) between effect size of expression and other molecular trait QTLs at overlapping signals (LD ≥0.8). (G) Fold-enrichment of eQTLs, hQTLs, and meQTLs in different chromatin segmentation states. See also Figures S2, S3, and S4 and Tables S2, S3, and S4.

We have shown that genetic variants determine a large fraction of observed epigenetic and transcriptional variation. Identifying these variants is essential to study their potential influence on cell function and disease mechanisms at individual loci. We first applied linear mixed models (Casale et al., 2015) to test associations of ∼7 M DNA sequence variants with gene expression quantified from total RNA sequencing. We considered variants within 1 Mb of gene bodies, for a total of 20,403 human genes that have a minimum of ten read counts in one of the cell types, including 13,245 (65%) protein-coding and 7,158 (35%) non-coding genes. Overall, 6,513 (39.3%), 5,845 (38.9%), and 5,799 (33.9%) genes had a QTL in monocytes, neutrophils, and T cells, respectively (2,482 non-coding genes; Figure 3A), encompassing biological functions that were for the most part separate to genes with uniquely epigenetic influences (Table S3).

We next sought to identify shared genetic effects linking genes to their putative regulatory elements such as gene promoters and enhancers. Enhancers play a central role in driving cell-type-specific gene expression (Ong and Corces, 2012), by activating transcription of target genes that may be located at distances of tens to hundreds or even thousands of kilobases. Here, we considered two different histone modifications typically associated with poised and active promoters and enhancers (H3K4me1 and H3K27ac), and DNA methylation levels measured using Illumina 450k arrays. We again tested associations by considering genetic variants within 1 Mb *cis*- windows centered on each feature. On average, 9.89% of methylation probes (64,836 probe-trait association), 25.7% of H3K4me1 peaks (21,829 peaks), and 11.5% of H3K27ac peaks (15,548 peaks) had at least one QTL associated with them. The majority of QTLs were associated with one phenotype (e.g., expression of one gene or one histone peak), while a fraction of them were linked to up to 15–80 phenotypes (Figure 3B).

Using the π1 statistics to assess the extent of sharing of genetic signals between the three immune types showed highly cell-type-specific effects at both histone modifications (H3K27ac, π1 = 0.27–0.44 and H3K4me1, π1 = 0.23–0.57; Figure 3C), consistent with predominantly cell-type-specific patterns of association for enhancers (Farh et al., 2015). Cell specificity was lower for expression quantitative trait loci (eQTLs) (π1 = 0.71–0.85) and methylation quantitative trait loci (meQTLs) (π1 = 0.79–0.93); sharing between the two myeloid cells was marginally greater than with T cells (Figure 3C). Across cell types, shared QTLs (defined by linkage disequilibrium [LD] r^2^ ≥ 0.8) had predominantly concordant direction of effect (i.e., the same allele similarly increased or decreased a trait in the two comparison cells; Figure 3D).

As shown earlier, genetic, epigenetic, and gene expression variance within a given cell type are strongly locally coordinated. We thus sought to identify shared genetic effects linking genes to their putative regulatory elements. We considered all eQTL sentinel variants (eSNPs) and asked whether the same variant was also associated with histone modification or methylation status. For this comparison, we required that the variant was either identical or in high LD (r^2^ ≥ 0.8) with the corresponding histone quantitative trait loci (hQTL) or meQTL sentinel variant. Using this rule, ∼43.3% of eSNPs were also associated with H3K4me1 or H3K27ac hQTLs (Figure 3E), denoting extensive local (median distance, 57 kb) coordination of genetic influences on gene expression and histone modifications. At shared variants, there was strong positive correlation of per-allele effect size between eQTLs and hQTLs at both histone marks (Figure 3F), indicating a predominant activating role. Approximately 43.3% of eQTL sentinel variants were also associated with a methylation probe, 44.2% of which within corresponding eGenes (Figure 3E). The effect sizes for these meQTLs were weakly negatively correlated to eQTLs of corresponding genes (Figure 3F) suggestive of chance overlap or a partial uncoupling between the two (Gutierrez-Arcelus et al., 2013). Further, QTLs mapped to distinct regulatory domains defined by chromatin states in matched cells (Carrillo de Santa Pau et al., 2016), where eQTLs were enriched at transcribed regions and transcription start sites (Figure 3G), meQTLs around Polycomb-repressed transcription start site (TSS) regions, and hQTLs at active enhancer and TSS states. The QTLs provide a rich catalog of putative regulatory elements for genes implicated in immune function (Table S4). We describe further examples in the context of allelic expression and disease-focused analyses. Overall, these results demonstrate that associations are highly coordinated within the three cells, and genetic effects underpin observed correlation between molecular traits.

### Genetic Regulation of Alternative Splicing

Alternative splicing regulates lineage commitment of human blood progenitors into mature blood cells (Chen et al., 2014) and contributes to disease as shown in lymphoblastoid cell lines (Li et al., 2016). We explored genetic influences to alternative mRNA splicing in the three primary immune cells using two complementary methods of quantification. In a first approach, we computed the ratio of alternatively spliced junctions (percent-splice-in or PSI) from mapped total RNA reads (Chen et al., 2014), allowing detailed surveys of splicing junctions for both annotated exons and also for exons annotated de novo from RNA-sequencing data in the three cell types. We identified a total of 32,357 alternatively spliced events (PSI) in 6,560 annotated genes and 2,288 unannotated transcripts. We then tested association with SNPs within 1-Mb regions surrounding each transcript, using comparable approaches to the eQTL analysis. As a second approach, we used sQTLseekeR to test for SNPs associated with variation in the relative abundance of a gene’s transcript isoforms (ISO). Here, we limited testing to associations for local (within the gene body ±5 kb) effects for annotated transcripts of protein-coding genes. In total, 9,485 genes and 1,462,663 SNPs were tested for association and FDR was used to correct for multiple testing. These two methods thus provide complementary analysis of alternative splicing, whereby ISO analysis is more sensitive to events involving main isoforms changes, while PSI is able to recover more subtle splicing patterns involving novel exons.

**Figure 4:**
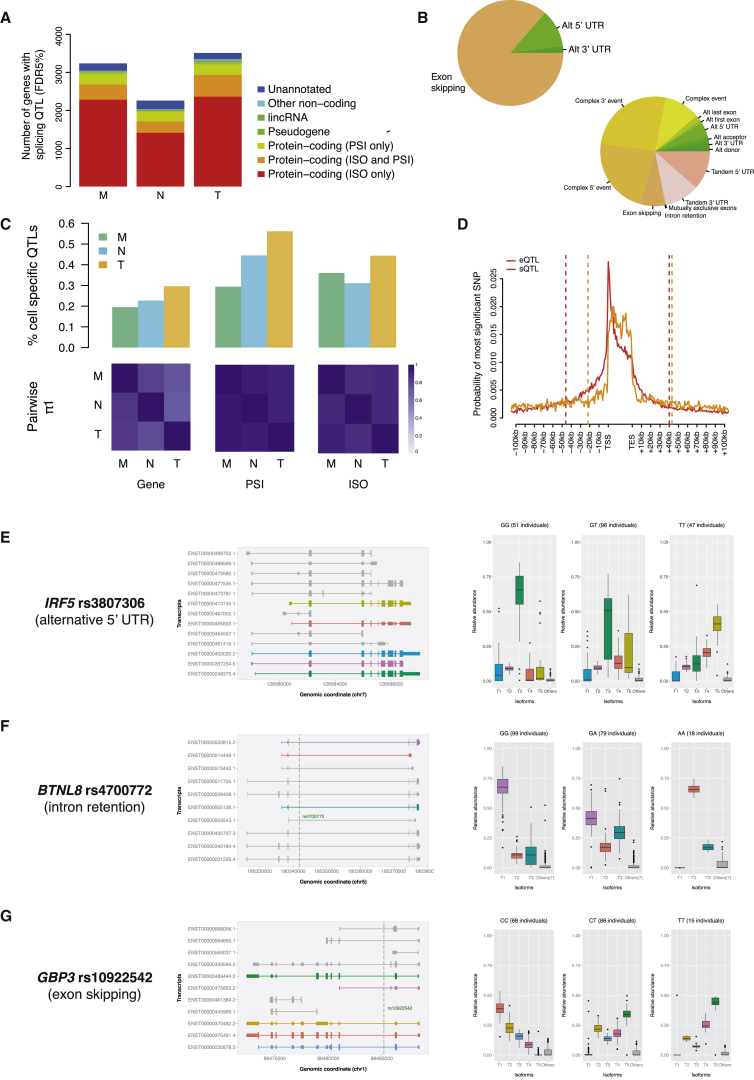
Features, Cell-type Specificity, and Examples of Splicing QTLs (A) Number of protein-coding, non-coding gene, and unannotated events with a significant splicing QTL (FDR <5%). (B) Percentage of different alternative splicing events from PSI (top) and ISO (bottom) analyses. (C) Percentage of PSI and ISO events that are cell-type-specific (top), and genome-wide patterns of QTL sharing (π1 statistics) among the three cell types (bottom). (D) Probability distribution of lead eQTL and sQTL SNPs around genes. (E–G) Examples of alternatively spliced genes showing transcript structure and their distribution based on genotypes at each ISO sQTL. (E) *IRF5* and rs3807306, a RA-predisposing SNP that is associated with the switch of two major isoforms that have alternative 5′ UTR in neutrophils. (F) *BTNL8* gene structure and rs47007720, which switches a protein-coding major isoform to a non-coding isoform with intron retention in neutrophils. (G) *GBP3* gene structure and rs10922542, which switches a protein-coding major isoform to a nonsense-mediated-decay isoform and involves an exon skipping event in T cells. See also Figure S3 and Tables S2, S3, and S4.

On average, QTLs were detected (FDR 5%) for 15.3% of PSI events and 33.2% of ISO events, corresponding to 18.4% of the genes tested. A sizeable fraction of PSI splicing quantitative trait loci (sQTLs) (9.6%–11.7%) involved non protein-coding genes (Figure 4A), suggesting alternative splicing of non-coding RNA species may provide an additional layer of genetic regulation of cellular identity and function. The number of sQTL genes (sGenes) was lower in neutrophils (2,260 and 15.0% of tested genes) compared to monocytes and T cells (Figure 4A), reflecting both lower levels of expression and higher rates of intron retention in neutrophils compared to the other cells (Wong et al., 2013). The majority of PSI QTLs involved exon-skipping events, followed by alternative 5′ or 3′ events, while the majority of ISO QTLs were complex events, given one isoform can contain several alternative splicing events (Figure 4B).

When considering alternative splicing events observed in two or more cells, the degree of sharing for sQTLs was higher than previously reported for eQTLs (π1 statistic = 0.88–0.96 for PSI and ISO; Figure 4C), and the effect sizes of shared sQTLs were highly correlated across cells (r = 0.94–0.97). However, a large proportion of sQTLs were specific to individual cell types (Figure 4C), for example, up to 56% of T cell PSI QTLs were for alternative splicing events that are only found in T cells. Overall, this suggests that although alternative splicing events tend to be highly cell-type-specific, genetic associations for alternative splicing events detected in two or more cell types are typically consistent. Genetic influences on splicing were predominantly independent from gene expression (Li et al., 2016), as reflected by predominantly unlinked eSNP-sSNP pairs (80% pairs with r^2^ < 0.1 within-cell) and the different distribution of eQTLs and sQTLs within genes (Figure 4D). Further, ISO QTLs were enriched closer to splice sites (averaging 1.9-fold) and the nearest exon (averaging 1.6-fold) when compared to non-ISO QTLs (p < 0.05, Fisher’s exact test). A subset of genetic variants was predicted to result in switch of major isoforms in genes of key importance in immunity and disease (Figures 4E–4G; Tables S3 and S4), potentially involving switch to non-coding transcripts, or nonsense-mediated decay.

### Allele-Specific Mapping from RNA-Sequencing and Histone Marks

**Figure S7:**
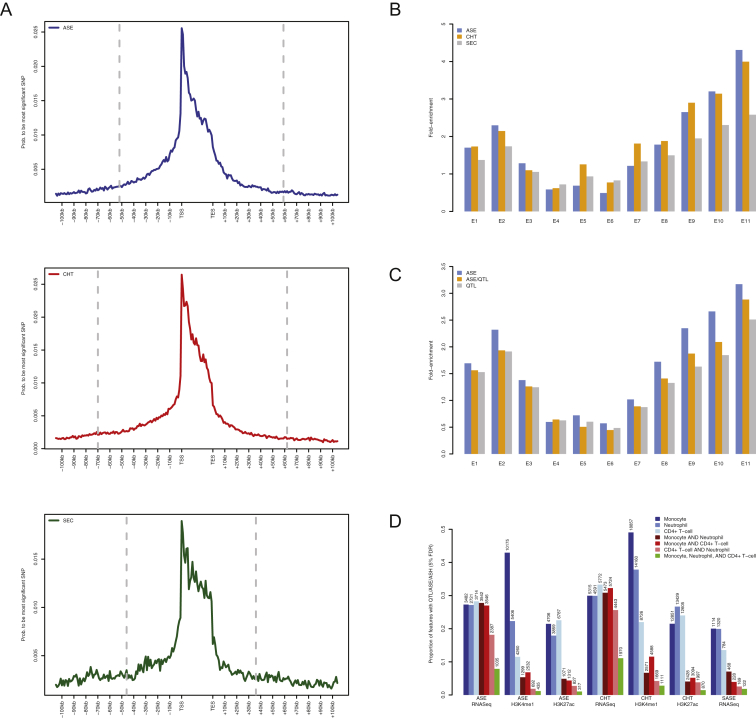
Distribution of Primary ASE Associations, Related to Figures 5 and 6 (A) Distribution of primary associations with respect to measured transcript for ASE (Blue), CHT (Red), or secondary, conditional ASE (Green) associations. The relative density of associations is adjusted to tested common SNPs in different bins. (B) Enrichment of chromHMM chromatin states for top primary ASE (Blue), primary CHT (Orange), or secondary ASE (gray) associations. The y axis is the fold-enrichment of SNPs in E1-E11 chromHMM states relative to all SNPs tested for association. (C) Enrichment of chromHMM chromatin states for primary ASE associations (Blue), top primary associations overlapping from ASE and QTL tests (Orange), and from QTL tests (gray). The y axis is the fold-enrichment of SNPs in E1-E11 chromHMM states relative to all SNPs tested for association. (D) Proportion of associations versus tested traits. For each type of test (ASE/CHE/ASES) and assay (Gene, H3K27ac, H3K4me1), the proportion of features with a QTL/ASE/ASH at 5% FDR relative to the total number of features tested is shown as bar graph for each cell type alone (Blue shade), common to two cell types (Red shade), and common all three cell types (green). The actual number of features at 5% FDR is shown above each bar.

As a complementary strategy to standard QTL mapping, we also considered allele-specific effects. By exploiting within sample variation, allelic analyses can help to identify *cis*-regulatory variation in the presence of strong confounding (e.g., *trans*-acting loci, non-genetic effects), as well as uncover rare and private regulatory variants (Pastinen, 2010). We chose an approach maximizing allelic information from total RNA-seq, summing up strand-specific allele counts across GENCODE v15 transcript regions (exons and introns) and removed reads with mapping biases. We applied two models for primary association tests either using allelic information alone (allele-specific expression mapping [ASE]) or in combination with read depth of non-allelic reads (combined haplotype test [CHT]). Overall, 74%–86% of genes showed significant ASE or CHT (FDR 5%) with common SNPs (MAF >5%). Using peak sets described for hQTL mapping, we also mapped allelic variation in H3K27ac and H3K4me1 signals by same approaches used for RNA-sequencing. Histone peaks showed lower mapping efficiency (20%–36% mapped using linear AS test or CHT, respectively) likely due to generally lower allelic information of shorter peak regions as compared to genes. Similar to QTL mapping, we observed stronger cell-type-dependence of allele-specific chromatin states as compared to ASE (Figure S7D).

Variants mapped by allelic tests showed large overlap with QTL-mapping, with 29%–43% top SNPs shared (r^2^ ≥ 0.8), with ASE showing slightly better specificity as compared to CHT (that has higher power). There was strong agreement (43%–58% of shared mapped lead associations) of ASE versus CHT associations across expression and chromatin traits. Both allelic approaches showed similar regulatory variant distributions in gene flanking regions as compared to QTL mapped variants (Figure S7A) and allele-specific associations tests show functional enrichments to expected chromatin states (Figures S7B and S7C). Altogether, these results indicate that each method captures additional true associations.

**Figure 5:**
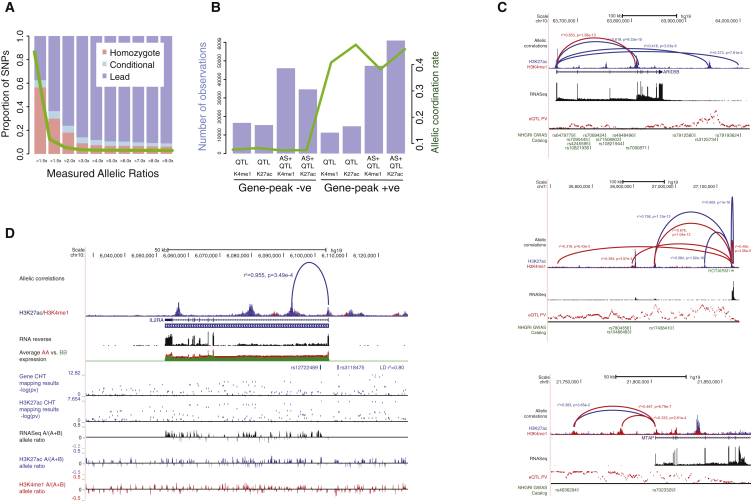
Features of Molecular Traits Revealed by Allelic Analyses (A) Relationship of significant allelic expression imbalance and mapped common *cis*-regulatory SNPs. Nearly 90% of transcripts show <1.5-fold difference between maternal and paternal copy (green line) with >2-fold differences seen in only ∼3% of transcripts. The primary (blue bar) or secondary (light blue) ASE mapped SNPs account for the majority of significant allelic effects, because homozygosity for these *cis*-rSNPs (red bars) is observed in only ∼7% cases with allelic imbalances >3-fold. (B) Coordinated genetic effects for genes and local chromatin peaks (lead SNPs r^2^ ≥ 0.8) are approximately four times more numerous (blue bars “Gene-peak +ve” AS+QTL) when both allelic and QTL mapping hits are considered as compared to QTL mapped hits alone (blue bars “Gene-peak +ve” QTL) and can be validated in up to 47% cases (green line) by intra-individual allelic correlation among genes and peaks. Genes with QTLs (QTL or AS+QTL) without coordinated genetic effects do not show (<5%) allelic correlation of local peaks. (C) Validated gene TSS/peak allelic coordination (arcs scaled by Pearson r^2^). Three (blue arc) H3K27ac and one H3K4me1 (red) elements linked allelicly to *ARID5B*, and similar allelic coordination for *MTAP*, while *HOTAIRM1* is linked to multiple regulatory elements. For *ARID5B* and *MTAP*, the underlying SNPs (red [-log10] p value track “eQTL Pv”) overlap a coordinated peak as well as a GWAS variant (green NHGRI GWAS catalog SNPs on bottom) linked to rheumatoid arthritis and nevus counts, respectively. (D) Disease locus functional phenotype captured solely in allele-specific analyses. *IL2RA* SNP (rs12722489) is associated with multiple sclerosis and Crohn’s disease and is the top SNP for a H3K27ac CHT event spanning the transcript (blue bar); the top *IL2RA* CHT SNP is in high LD (r^2^ = 0.8) with the chromatin allelic signal. Allelic variations between gene and H3K27ac among individuals are extremely highly correlated (Pearson r^2^ > 0.95, blue arc), suggesting that allelic chromatin altered by disease SNP can lead to differential allelic expression of *IL2RA*. See also Figure S7 and Table S6.

We also applied allelic mapping to gain further insight into several features of mapped QTLs. Focusing on genes with deep-phased read measurements, we first estimated the proportion of allelic variation captured by primary ASE mapping. We also carried out conditional (secondary) ASE mapping among genes with FDR <5% primary ASE signal after removing samples heterozygous for SNP to uncover more common SNP effects and included these in our estimation of common SNP effects. Remarkably, over 90% of the differences of 3-fold or greater between allelic transcripts were captured by our mapped common SNPs; even lowering the threshold to 1.5-fold difference, we still observed >70% contribution of common SNPs (Figure 5A). These results indicate a predominant role for common SNPs in governing allelic traits and argue for the comprehensiveness of our catalog for assessing the *cis-*acting impact of common SNPs in these cells as well as set the upper boundary for prevalence of rare high-effect regulatory alleles.

Utilizing the complementary information of QTL and allelic mapping tests, we linked genes to regulatory elements using the strict LD criteria as previously done for QTLs (lead-SNP r^2^ ≥ 0.8). Joint use of all mapping approaches quadrupled genetically controlled gene-peak pairs offered by QTL mapping alone. Overall, 70%–89% of expression traits could be linked to at least one H3K4me1 or H3K27ac peak, respectively. Rich local genetic connectivity uncovered by joint allelic and QTL mapping were further validated by systematically correlating intra-individual allelic state of peaks and linked genes (Figure 5B). The shared allelic states provide orthogonal information complementing physical interaction maps and allow assessment of gene distal genetic effects often observed in association-mapping of disease SNPs (Figures 5C and 5D).

**Figure 6:**
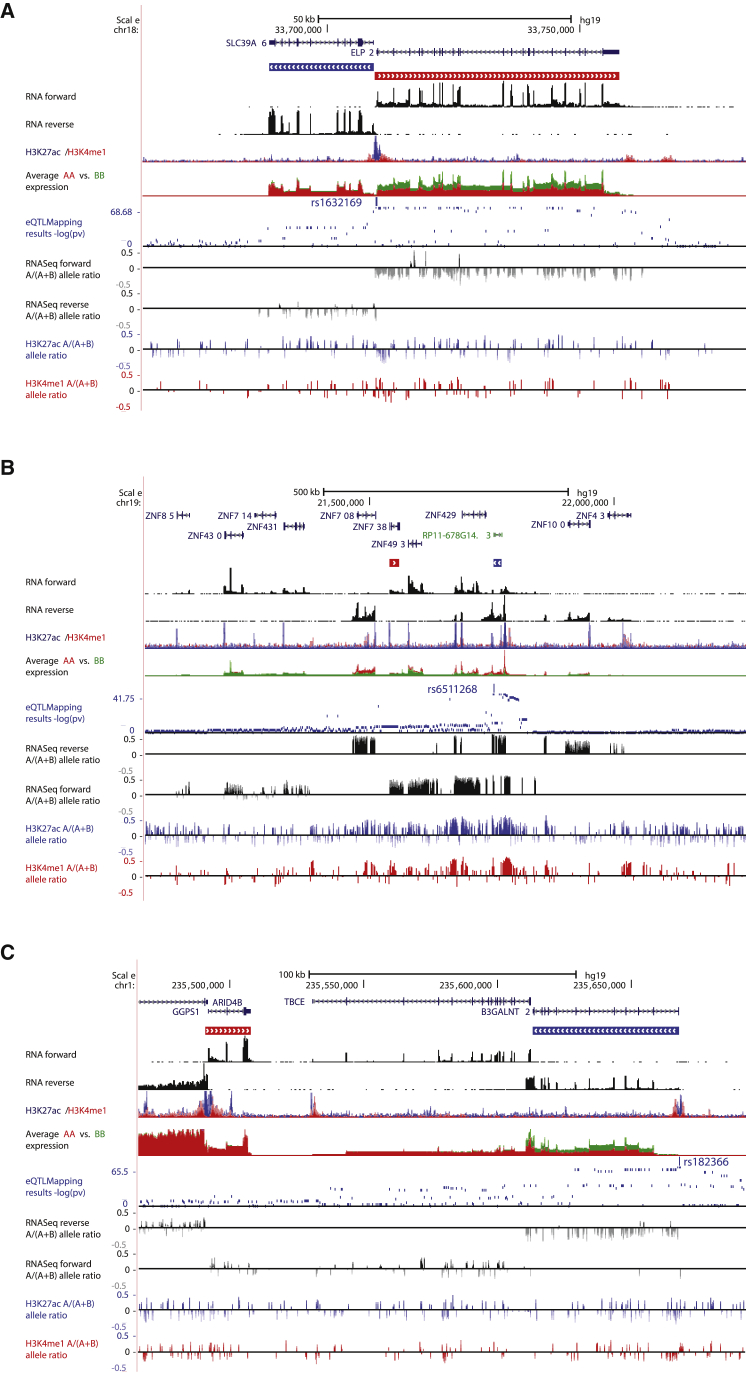
Allelic Behavior of Locally Correlated eQTLs Examples of modes for clustering of “cis-eQTLs.” Top to bottom: gene annotations (blue), eQTL pair sharing same top association (blue and red rectangles), local RNA-signal (fwd and rev strand; black), H3K4me1 (red) and H3K27ac (blue), average (log) RNA-seq intensity among top SNP eQTL SNP homozygotes (AA, red; BB, green), top SNP (blue tick and rsID), eQTL mapping result (-log10 p value track in blue), allelic expression deviation (equal expression = 0, monoallelic expression = |0.5|) among top QTL SNP heterozygotes in forward (black) and reverse (gray) strands, allelic H3K27ac (blue), and H3Kme1 (red) deviation among top QTL SNP heterozygotes. (A) “head-to-head” configuration of eQTL and allelic effect, where total and allelic difference is mapped to a variant in a bidirectional promoter. (B) local SNP altering both chromatin and reverse and forward strands across multiple transcripts and chromatin signal. (C) example of a putative “*cis*-*trans*” pair where *B3GALNT2* shows strong overexpression of one genotype and consistent allelic effect with eSNP localizing to its promoter, which also alters expression level of *GGPS1* without detectable allelic effect. See also Figure S7 and Table S6.

Finally, we explored modes of locally strongly correlated (sharing lead associations in LD r^2^ ≥ 0.9) expression QTLs, where genomic coordinates of transcripts were not overlapping, using allelic data. We identified 2,691 local eQTL pairs when limiting to intergene distance of 250 kb or less. The majority of these associations (59%) occurred in transcripts transcribed from the same strand (shared orientation); next, commonly (26%) the genes shared 5′ intergenic region (“head-to-head” orientation); in the rarest cases (15%) the genes sharing same SNP association were “tail-to-tail” orientation (sharing 3′ intergenic region). The distribution of locally shared associations deviated strongly from the null distribution (chi-square test = 71, 2 degrees of freedom [df]) (based on orientation of genes tested) where the strand sharing was more common (+12%) and “tail-to-tail” configuration was depleted (−31%). This can partly be explained by 5′ overall bias of regulatory variants giving rise to, for example, bi-directional promoter variants (Figure 6A, top). We also identified strong locally correlated allelic effects spanning multiple independent annotations, extending to chromatin layer and multiple genes in both strands (Figure 6B, middle). Perhaps the most intriguing associations were those where only one of the transcripts in the pair showed allelic expression effect with mapped local QTL, whereas other transcripts showed equal allelic expression for same eSNP (Figure 6C, bottom). This could indicate local *trans* acting activity of the verified *cis* variant. This hypothesis was supported in follow-up analyses where we tested 342 “*cis*-eQTLs” showing potential local *cis* and *trans* effects (the latter showing no allelic bias despite high allelic informativity) for genome-wide *trans*-associations and compared them to control set of 678 lead eSNPs (matched by mapping significance and distance from TSS). The candidate local *trans*-activators showed 2-fold enrichment (p = 0.0003, Fisher’s exact test) of *trans*-associations in different chromosomes as compared to matched control eQTLs (14% versus 7% of SNPs with *trans*-associations FDR <5%) establishing that allelic information can be utilized to reveal unexpected features in local eQTLs.

### Mapping Molecular Mechanisms at Disease-Associated Variants

**Figure 7:**
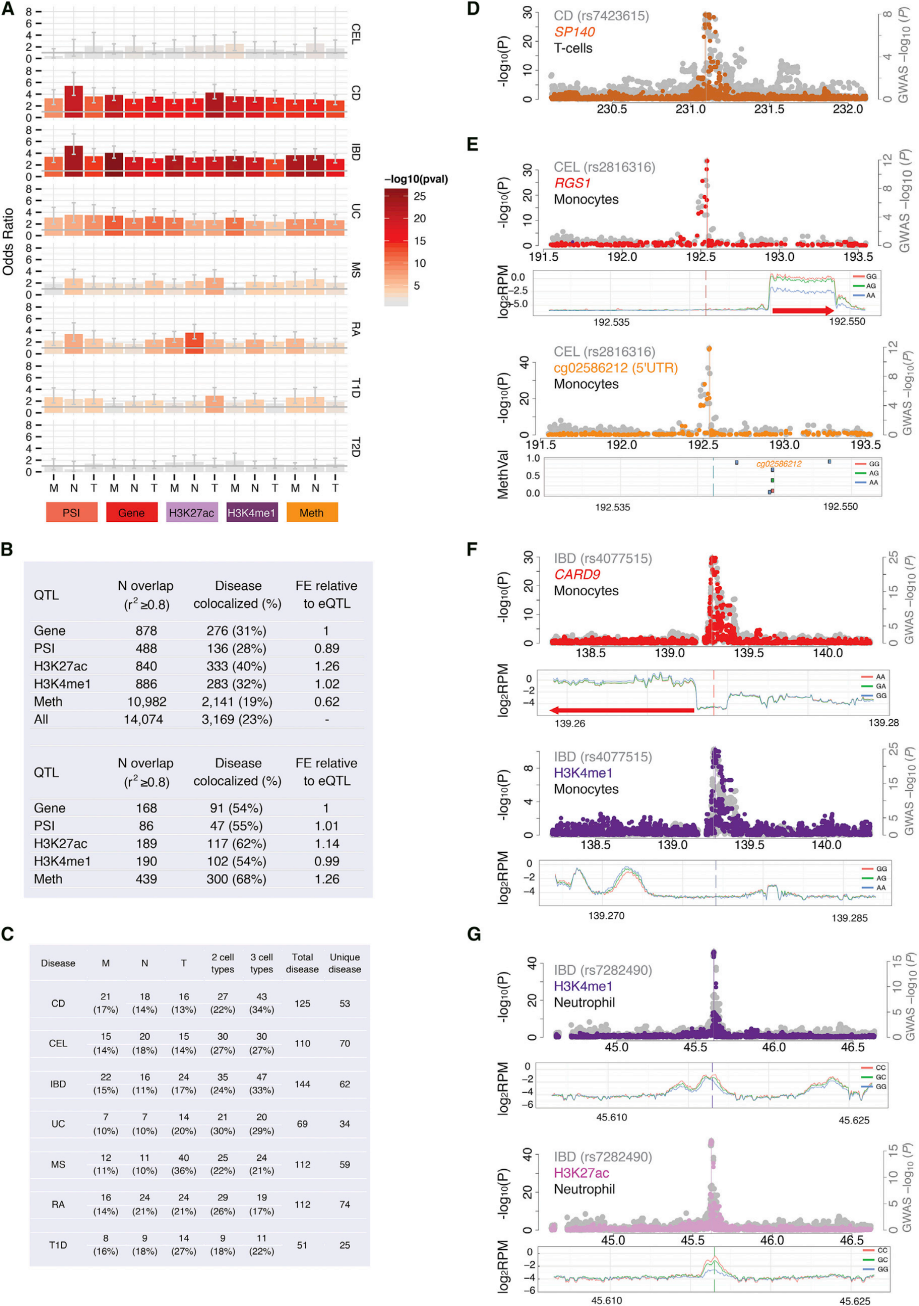
Molecular Mechanisms at Autoimmune Disease Loci (A) Enrichment in molecular QTLs of celiac disease (CEL), Crohn’s disease (CD), inflammatory bowel disease (IBD), ulcerative colitis (UC), multiple sclerosis (MS), rheumatoid arthritis (RA), and type 1 (T1D) and type 2 diabetes (T2D). (B) N overlap = Number of observed QTL-trait pairs (top table) or unique disease loci (bottom table) that overlap (r^2^ ≥ 0.8) disease variants across all three cell types. Disease colocalized = number and proportion of overlapping pairs that colocalize with disease variants with PP3 ≥ 0.99. FE = Ratio of fold enrichment of these proportions over eQTLs. (C) Number (%) of disease loci colocalizing with cell-type-specific molecular QTLs, for associations unique to M, N, T, or shared between two or three cell types. (D–G) Examples of colocalization between disease and molecular traits. Each plot shows regional association (window 2 Mb centered on the significant peak) for a given disease locus (gray), molecular mark (color) and cell type, and corresponding molecular trait signal coverage (log2 RPM, 20 kb). (D) PSI sQTL. (E) eQTL/meQTL. (F) eQTL/hQTL. (G) hQTL with no corresponding eQTL. See Table S5.

Many GWAS loci map to regulatory domains. Thus, a key goal of this study was to explore the value of molecular trait associations to annotate putative functional consequences of disease-associated loci with single-base resolution. We focused on seven autoimmune diseases (celiac disease [CEL] [Dubois et al., 2010], inflammatory bowel disease [IBD] [Liu et al., 2015], including Crohn’s disease [CD] and ulcerative colitis [UC], multiple sclerosis [MS] [Beecham et al., 2013], type 1 diabetes [T1D] [Onengut-Gumuscu et al., 2015], and rheumatoid arthritis [RA] [Okada et al., 2014]), for which we retrieved publicly available genome-wide summary statistics. We first tested genome-wide enrichment for variants nominally associated with disease (p value ≤ 10^−5^), applying an enrichment test controlling for LD, local gene density, and variant minor allele frequency (Iotchkova et al., 2016). We detected moderate-to-strong enrichment of disease associations for all classes of molecular QTLs tested and specific to autoimmune diseases with limited evidence for cell-type specificity of enrichments (Figure 7A).

These significant overlaps suggest these are not chance events, motivating to investigate individual co-localized variants. To identify such loci, we first systematically intersected (LD r^2^ ≥ 0.8) disease-associated variants with molecular QTLs and identified 14,074 instances of trait-locus overlap (Figure 7B). We then applied a more stringent Bayesian model test to estimate the posterior probability (PP) of each genomic locus containing a single variant affecting both disease and molecular trait (“colocalization”) against other possible models (single trait or two independent associations) (Giambartolomei et al., 2014, Pickrell et al., 2016). Of note, the colocalization model does not differentiate a causal relationship between a molecular trait and disease from independent (“pleiotropic”) effects driven by the same. Further, in regions of extended LD, the model has limited power to distinguish colocalization from two variants in high LD but with independent effects on phenotype.

Overall, 3,169 disease-molecular trait pairs (or 23%) had high posterior probability for colocalization according to a stringent cutoff (PP3 ≥ 0.99), corresponding to 345 unique disease loci (Figures 7B and 7C; Table S5). Colocalization of H3K27ac marks displayed marginally higher levels of enrichment within the colocalized set compared to eQTLs after accounting for the overall number of overlapped loci in each mark (Fisher’s p value < 0.01; Figure 7B). MS, T1D, and UC had associations predominantly colocalizing with T cell marks, compared to other diseases (Figure 7C).

These overlaps offer insights into disease associations and disease specificity, (Figures 7D–7G; Table S5). Colocalization to eQTLs/sQTLs (31% of disease loci) guide the identification of possible functional gene candidates and mechanisms. For instance, CD associations centered on rs7423615 were colocalized with alternative splicing signal for *SP140* (nuclear body protein) in T cells (Figure 7D). Similarly, the MS-associated variant rs1800693 colocalizes with tumor necrosis factor receptor superfamily member 1A (*TNFRSF1A*) alternative splicing in monocytes and neutrophils; the rs917997 CEL variant colocalizes with interleukin 18 receptor accessory protein (*IL18RAP*) alternative splicing in neutrophils, rs35260072 (IBD) colocalizes with interferon regulatory factor 1 (*IRF1*) splicing in neutrophils; and the rs12936409 IBD/CD variant (Anderson et al., 2011) colocalizes with gasdermin B (*GSDMB*) splicing in T cells.

Intersection with DNA methylation or/and histone modifications allows extending mechanistic hypotheses for eQTLs (Figures 7E and 7F). For instance, the IBD/CD locus colocalized an eQTL rs1081768 that is associated with *TNFSF15* expression levels in monocytes and also with H3K27ac/H3K4me1 in the same cell. Similarly, IBD/CD SNP rs4077515 colocalized an eQTL governing *CARD9* expression that is also an hQTL for H3K4me1 in both monocytes and neutrophils (Figure 7F). Allele-specific analyses confirmed that H3K4me1 variation was linked to *CARD9 cis-*regulation in both cell types, suggesting weaker marginal effect due to perhaps lower activity of the affected enhancer in neutrophils was missed in eQTL mapping.

At least two thirds of disease-colocalized loci involved a DNA methylation or histone modification QTL without a corresponding eQTL, indicating a possible effect on poised or primed promoters/enhancers with no effect on gene expression levels at baseline conditions (while for a subset of loci incomplete ascertainment of eQTLs due to power limitations cannot be ruled out). An example is shown in Figure 7G, where the IBD/CD/UC variant rs7282490 (21q22.3) colocalized with H3K27ac and H3K4me1 variation, but not gene expression, in neutrophils. Associations at this locus were driven by rs8134436, mapping to within the two histone modification peaks. This SNP is also predicted to affect binding of pioneer transcription factors *PU1* and *CEBPB* in neutrophils (S.W., unpublished data), sits within an active enhancer chromatin state in neutrophils, and overlaps binding sites of multiple transcription factors (cohesin subunit *RAD21*, *CEBPB/E*, and *P300*) in the neutrophilic cell line HL60. Additional disease links similar to *CARD9* locus were evident from allele-specific analyses with ∼22% of histone mark or gene expression traits linked to disease SNPs observed solely in allele-specific datasets (Figure 5D).

Overall, these findings confirm the occurrence of widespread genetic regulation of immune and host defense pathways overlapping disease loci and involving not only gene expression but also splicing and epigenetic modifications. The occurrence of potential nonfunctional (chance) overlap at individual loci will require careful follow-up studies to validate functional hypotheses. Nevertheless, these results suggest the convergence of independent regulatory layers for cell-specific function, as well as independent techniques for their measurement, yields biological validity to mapped traits well beyond traditional eQTL studies.

## Discussion

We generated a high quality expansive resource for the scientific community. Exploiting this unprecedented dataset, three distinct aspects of the interplay of genetic and epigenetic factors in gene regulation were explored. Variance decomposition analysis was used to obtain a quantitative assessment of the contribution of epigenetic factors to transcription, independent of *cis*-genetic influences. We showed that *cis*-genetic effects explain the majority of transcriptional variance for a majority of genes with relatively modest independent epigenetic influences for a small subset of biologically relevant genes. These results strongly suggest the need to adequately control for the effect of *cis*-genetic variation in epigenome-focused explorations. While our data only covers a fraction of epigenomic space (chromatin states, interactions, methylome) these observations are important in context of EWAS, which typically survey a smaller fraction of epigenome. In fact, our estimates may be conservative for the role of DNA sequence governing gene expression variance, because it is expected that *trans*-effects and rare *cis*-genetic effects will account for part of the *cis*-variance we attribute to independent epigenetic effects.

The use of allele-specific analysis in parallel with QTL mapping allowed us to expand the spectrum of genetic influences assayed in this study. True *cis*-regulatory variation is expected to give rise to allelic differences in distribution of sequence reads from functional elements (Pastinen, 2010) and can be used in “*cis-*rSNP” mapping (Adoue et al., 2014) with improved specificity when methods to tackle alignment biases leading to spurious signals (Kumasaka et al., 2016) are applied. The majority of coordinated, genetically controlled regulatory element connections require the combined discovery power of QTL and AS-specific mapping techniques, and thus our resource will allow detailed investigation of long-range interactions in context of population variation. We further demonstrate that large-magnitude allelic imbalance is rare and predominantly (up to 93%) explained by common *cis-*regulatory variants. Finally, clustering of “*cis-*eQTLs” observed by us and earlier studies were explored, and diversity of mechanisms were suggested by allelic data, including bi-directional promoters, locally expanding chromatin effects, as well as local “*cis*-*trans*” pairs.

The use of high-resolution genome, transcriptome, and epigenome sequencing reveals genetic influences at disease variants captured by effects across epigenomic data layers, and the use of distinct primary immune cell lineages reveals a sizeable fraction of genetic variants where correlations are only visible in cell-specific contexts. These cases include hundreds of autoimmune disease variants likely acting through perturbation of local regulatory circuitry. Overlap between molecular and disease associations alone does not provide proof of causality. However, our rigorous approach combining strict linkage disequilibrium thresholds and statistical colocalization techniques yielded high value targets for experimental follow-up by the community to identify causal mechanisms.

Overall, the data and results are expected to improve understanding of the regulation of the transcriptional machinery in three important cells of the immune system. This deep characterization of molecular events is expected to substantially boost focused functional explorations of human disease variants, revealing potential new disease mechanisms and therapeutic opportunities.

## STAR★Methods

### Key Resources Table

**Table:** 

REAGENT or RESOURCE	SOURCE	IDENTIFIER
**Antibodies**		

CD16 microbeads	Miltenyi	Cat# 130-045-701
CD14 microbeads	Miltenyi	Cat# 130-050-201
MφP9 FITC	BD Biosciences	Cat# 345786
B73.1 / leu11c PE	BD Biosciences	Cat# 347617
VEP13, MACS PE	Miltenyi	Cat# 130-091-245
BIRMA 17C FITC	IBGRL-NHS	Cat# 9453CE
RPA-T4 FITC	BD Biosciences	Cat# 561842
HI100 PE	BD Biosciences	Cat# 555489
H3K4me1	Diagenode	Cat# C15410194
H3K27ac	Diagenode	Cat# C15410196

**Critical Commercial Assays**		

EasySep Human Naive CD4+ T Cell Enrichment Kit	StemCell	Cat# 19155
Illumina TruSeq Stranded Total RNA Kit with Ribo-Zero Gold	Illumina	Cat# RS-122-2201
DNeasy Blood & Tissue Kit	QIAGEN	Cat# 69506
EZ-96 DNA Methylation MagPrep Kit	Zymo Research	Cat# D5040
Infinium HumanMethylation450 assays superseded by Infinium MethylationEPIC BeadChip Kit	Illumina	Cat# WG-317-1001
Qiaquick MinElute PCR purification Kit	QIAGEN	Cat# 28004
Kapa Hyper Prep Kit	Kappa	Cat# KK8500
Agencourt AMPure XP	Agencourt	Cat# A63880
Protein A Dynabeads	Invitrogen	Cat# 10001D
NEBnext	New England Biolabs	Cat# E6000S
Ideal Kit	Diagenode	Cat# C01010011
GeneRead Size Selection kit	QIAGEN	Cat# 180514

**Deposited Data**		

1000 Genomes Project		http://www.1000genomes.org/data/
		ftp://ftp.1000genomes.ebi.ac.uk/vol1/ftp/
GENCODE 15	Harrow et al., 2012	http://www.gencodegenes.org/releases/15.html
ENCODE blacklisted regions		http://hgdownload.cse.ucsc.edu/goldenPath/hg19/encodeDCC/wgEncodeMapability/wgEncodeDacMapabilityConsensusExcludable.bed.gz
GWAS Catalog	Welter et al., 2014	https://www.ebi.ac.uk/gwas/
Blueprint GRCh37 genome and gene annotation	N/A	ftp://ftp.ebi.ac.uk/pub/databases/blueprint/releases/20130301/homo_sapiens/reference
WGS data files	This paper	EGAD00001002663
RNA data files	This paper	EGAD00001002671
		EGAD00001002674
		EGAD00001002675
ChIP-seq data files	This paper	EGAD00001002670
		EGAD00001002672
		EGAD00001002673
450k data files	This paper	EGAS00001001456

**Software and Algorithms**		

BWA (v0.5.9)	Li and Durbin, 2009	http://bio-bwa.sourceforge.net/
Picard (v1.98)	N/A	https://github.com/broadinstitute/picard
GATK (v3.4)	DePristo et al., 2011	https://www.broadinstitute.org/gatk/download/auth?package=GATK-archive&version=3.4-0-g7e26428
SAMtools/bcftools	Li, 2011	https://github.com/SAMtools/SAMtools/releases/tag/1.2
VQSR	DePristo et al., 2011	https://www.broadinstitute.org/gatk/guide/article?id=39
BEAGLE r1398	Browning and Browning, 2007	https://faculty.washington.edu/browning/beagle/
PLINK v1.9	Purcell et al., 2007	https://www.cog-genomics.org/plink2
FastQC (v0.10.1)	N/A	http://www.bioinformatics.babraham.ac.uk/projects/fastqc/
STAR (v2.4.0k)	Dobin et al., 2013	https://github.com/alexdobin/STAR
DESeq2 (v1.4.5)	Love et al., 2014	https://bioconductor.org/install/#install-bioconductor-packages
Cufflinks (v2.2.1.)	Trapnell et al., 2010	http://cole-trapnell-lab.github.io/cufflinks/releases/v2.2.1/
sQTLseekeR R package (v2.0)	Monlong et al., 2014	https://github.com/jmonlong/sQTLseekeR
AStalavista 3.2	Foissac and Sammeth, 2007, Monlong et al., 2014	http://sammeth.net/confluence/display/ASTA/2+-+Download
Minfi	Aryee et al., 2014	https://bioconductor.org/packages/release/bioc/html/minfi.html
NOOB	Triche et al., 2013	https://www.bioconductor.org/packages/release/bioc/html/methylumi.html
SWAN	Maksimovic et al., 2012	https://bioconductor.org/packages/release/bioc/html/minfi.html
SVA	Leek et al., 2012	http://www.bioconductor.org/packages/release/bioc/html/sva.html
PhantomPeakQualTools vr18	N/A	http://code.google.com/p/phantompeakqualtools/
MACS2 v2.0.10.20131216	Zhang et al., 2008	https://pypi.python.org/pypi/MACS2
BEDOPS v2.4.14	N/A	http://bedops.readthedocs.io/en/latest/
ComBat	Chen et al., 2011	http://www.bioconductor.org/packages/release/bioc/html/sva.html
PEER	Stegle et al., 2012	https://github.com/PMBio/peer/wiki
DNA Methylation Age Calculator	Horvath, 2013	https://dnamage.genetics.ucla.edu/
LIMIX	Casale et al., 2015, Lippert et al., 2014	https://github.com/PMBio/limix
association testing (EMMA)	Kang et al., 2008a	http://mouse.cs.ucla.edu/emma/
multiple hypothesis correction (LRVM)	Battle et al., 2014	http://dags.stanford.edu/dgn/
heritability analysis (GCTA)	Yang et al., 2011	http://cnsgenomics.com/software/gcta/
Pysam	N/A	https://github.com/pysam-developers/pysam
WASP	van de Geijn et al., 2015	https://github.com/bmvdgeijn/WASP
qvalue (v1.99.1)	Storey and Tibshirani, 2003	https://github.com/jdstorey/qvalue
GARFIELD	N/A	http://www.ebi.ac.uk/birney-srv/GARFIELD/
		http://bioconductor.org/packages/release/bioc/html/garfield.html
ChromHMM	Ernst and Kellis, 2012	http://compbio.mit.edu/ChromHMM/
Trim Galore v0.32	N/A	http://www.bioinformatics.babraham.ac.uk/projects/trim_galore/
Bedtools2 v2.23.0	N/A	https://github.com/arq5x/bedtools2/releases/tag/v2.23.0
gwas-pw	Pickrell et al., 2016	https://github.com/joepickrell/gwas-pw
Ingenuity Pathway Analysis (IPA) QIAGEN Redwood City	N/A	www.qiagen.com/ingenuity

### Contact for Reagent and Resource Sharing

Further information and requests for reagents may be directed to the corresponding author/lead contact; Nicole Soranzo (ns6@sanger.ac.uk).

### Experimental Model and Subject Details

#### Human Subjects

Blood was obtained from donors who were members of the NIHR Cambridge BioResource (http://www.cambridgebioresource.org.uk/) with informed consent (REC 12/EE/0040) at the NHS Blood and Transplant, Cambridge. Details of donor characteristics (gender, smoking status past and present and age bin), identification (ID) code and donation date are listed in Table S1. Blood collection is described in the STAR Methods.

### Method Details

#### Sample Collection and Cell Isolation

##### Peripheral Adult Blood Collection

Donors were on average 55 years old (range 20-75 years old) with 46% of donors being male. A unit of whole blood (475 ml) was collected in 3.2% Sodium Citrate. An aliquot of this sample was collected in EDTA for genomic DNA purification. A full blood count (FBC) for all donors was obtained from an EDTA blood sample, collected in parallel with the whole-blood unit, using a Sysmex Haematological analyzer. The level of C-reactive protein (CRP), an inflammatory marker, was also measured in the sera of all individuals. All donors used for the collection had FBC and CRP parameters within the normal healthy range. Blood was processed within 4 hr of collection.

##### Isolation of Cell Subsets

To obtain pure samples of ‘classical’ monocytes (CD14+ CD16-), neutrophils (CD66b+ CD16+) and naive CD4+ T cells (CD4+ CD45RA+), we implemented a multi-step purification strategy. Whole blood was diluted 1:1 in a buffer of Dulbecco’s Phosphate Buffered Saline (PBS, Sigma) containing 13mM sodium citrate tribasic dehydrate (Sigma) and 0.2% human serum albumin (HSA, PAA) and separated using an isotonic Percoll gradient of 1.078 g/ml (Fisher Scientific). Peripheral blood mononuclear cells (PBMCs) were collected and washed twice with buffer, diluted to 25 million cells/ml and separated into two layers, a monocyte rich layer and a lymphocyte rich layer, using a Percoll gradient of 1.066 g/ml. Cells from each layer were washed in PBS (13mM sodium citrate and 0.2% HSA) and subsets purified using an antibody/magnetic bead strategy. To purify monocytes, CD16+ cells were depleted from the monocyte rich layer using CD16 microbeads (Miltenyi) according to the manufacturer’s instructions. Cells were washed in PBS (13mM sodium citrate and 0.2% HSA) and CD14+ cells were positively selected using CD14 microbeads (Miltenyi). CD4+ naive T cells were negatively selected using an EasySep Human Naive CD4+ T Cell Enrichment Kit (StemCell) according to the manufacturer’s instructions. To purify neutrophils, the dense layer of cells from the 1.078 g/ml Percoll separation was lysed twice using an ammonium chloride buffer to remove erythrocytes. The resulting cells (including neutrophils and eosinophils) were washed and neutrophils positively selected using CD16 microbeads (Miltenyi) according to the manufacturer’s instructions. The purity of each cell preparation was assessed by multicolor FACS (Figure S1) using conjugated antibodies for CD14 (MφP9, BD Biosciences) and CD16 (B73.1 / leu11c, BD Biosciences) for monocytes, CD16 (VEP13, MACS, Miltenyi) and CD66b (BIRMA 17C, IBGRL-NHS) for neutrophils and CD4 (RPA-T4, BD) and CD45RA (HI100, BD) for naive CD4+ T cells. Purity was on average 95% for monocytes, 98% for neutrophils and 93% for naive CD4+ T cells.

#### Molecular Data Generation and Processing

##### Genome and Annotation Version

All alignments and analyses in the Blueprint EpiVar project were carried out using GRCh37/hg19 and GENCODE 15 (Harrow et al., 2012).

##### Whole-Genome Sequencing

*Sample Preparation.* Genomic DNA preparation was performed at the University of Cambridge (UCAM). Red blood cells from EDTA whole blood were lysed prior to lysis of leukocytes using guanidine hydrochloride, sodium acetate and a protease lysis buffer. DNA was extracted using chloroform and precipitated in ethanol prior to washing, resuspension in ultra-pure water and quantification (Qubit, Invitrogen).

*Library Preparation.* Whole-genome sequencing (WGS) was performed at the Wellcome Trust Sanger Institute (WTSI). Genomic DNA (approximately 1μg) was fragmented to an average size of 500 base pairs (bp), and indexed, adaptor-ligated DNA libraries were created using established Illumina paired-end protocols. A portion of each library was used to create an equimolar pool comprising of eight indexed libraries.

*Sequence Data Generation*. Libraries were subjected to 100bp paired-end (PE) sequencing (HiSeq 2000/2500; Illumina) at the WTSI following manufacturer’s instructions. Each pool of eight libraries was sequenced on multiple lanes/flowcells to an (average) depth of 7.05x coverage (SD = 1.84) of the human genome and aligned to GRCh37/hg19 using BWA (v0.5.9) (Li and Durbin, 2009).

*Sequence Data Processing*. Sequence data were processed by the Human Genetics Informatics Group at the WTSI as described in more detail in Gurdasani et al. (2015). Briefly the following steps were carried out:

BAM Processing. After creating BAM files from the sequenced lanes, base qualities were recalibrated (Abnizova et al., 2010) and mapped to the human reference genome (GRCh37/hg19) with BWA. BAM files were sorted and duplicates were marked using Picard (v1.98). Then BAMs were realigned around known and discovered INDELs using GATK (v3.4)) (DePristo et al., 2011) and re-calibrated by GATK.
Variant Calling. SNP and INDEL calls were made using SAMtools/bcftools (Li, 2011) by pooling the alignments from 200 individual low coverage BAM files. All-samples and all-sites genotype likelihood files (bcf) were created with SAMtools mpileup on chunked chromosomes. The resulting VCFs were merged and Variant Quality Score Recalibration (VQSR) (DePristo et al., 2011) was performed on the chunks, independently for SNPs and INDELs. GATK was run independently for SNPs and INDELs producing a VCF file containing variant quality score log odds ratio (VQSLOD) scores for each site. The VQSR filter was applied to the SAMtools calls.
Variant Quality Control and Filtering. We filtered variants that were identified as an INDEL within 10 bp of an INDEL and a SNP within 3 bp of an INDEL. Additionally, variants were filtered if their VQSLOD score was below the score that was necessary to discover 96% of truth sites. For SNPs this cut-off was a minimum VQSLOD score of 1.0078 and for INDELs a score of 0.91. The missing and low confidence genotypes in the filtered VCFs were filled in with BEAGLE r1398 (Browning and Browning, 2007). Additional filtering was then applied to generate a final dataset containing variants with (i) Allelic R-Squared (AR2) ≥ 0.8 (AR2 is the estimated squared correlation between the most likely allele dosage and the true allele dosage); (ii) Hardy-Weinberg equilibrium (HWE) ≥ 1x10^−3^; and (iii) allele count (AC) > 4.

*Data QC.* A set of 154,222 robustly QC’d autosomal SNPs extracted from a total of 7,009,917 was used to carry out sample quality control using principal components analysis (PCA) for the identification of ethnic outliers and Identity-By-Descent (IBD) analysis for the identification of duplicate samples. The SNPs used for the sample quality control consisted of bi-allelic variants with minor allele frequency (MAF) ≥ 0.05, Hardy-Weinberg P value ≥ 10^−4^ and genotype missingness < 3%. In addition, a pairwise *r*^*2*^ threshold of 0.2 was used to select unlinked SNPs. This was done using the *indep-pairwise* function within PLINK v1.9 (Purcell et al., 2007), with a moving window of 1000bp. Ethnicity was evaluated by merging the BLUEPRINT samples with the 14 populations present in the 1000 Genomes Project data. PCA was performed and the first three principal components were plotted to identify possible ethnic outliers (see Figure S2A). A threshold on PC2 scores of −0.018 was used to differentiate the samples of European origin (GBR, CEU, TSI, FIN, IBS) from the rest. In total 3 outliers were identified and excluded as being of mixed ethnic origins. The proportion of alleles that were IBD was estimated in a pairwise manner for all samples using the PLINK Method-of-Moments function. The probability of sharing zero alleles by descent was found to be between (Z0) 0.91 and 1 for all pairwise estimations and therefore all the individuals in the data were defined as unrelated. Other metrics for the complete variant call set, such as number of variants per sample and allele frequency, as well as depth of coverage and Ts/Tv ratio, are shown in Figures S2B–S2H.

##### RNA-Sequencing Sample Preparation

RNA sequencing (RNA-seq) preparation and library creation at McGill University (naive CD4+ T cells) and the Max Planck Institute for Molecular Genetics (MPIMG, monocytes and neutrophils) were performed using identical methods. Following purification, cells were lysed in TRIZOL reagent (Life Technologies) at a concentration of approximately 2.5 million cells/ml. RNA was extracted as per manufacturer’s instructions, resuspended in ultra-pure water and quantified (Qubit, Invitrogen) prior to library preparation.

##### Data Generation

*Library preparation.* Sequencing libraries were prepared from 200ng RNA using an Illumina TruSeq Stranded Total RNA Kit with Ribo-Zero Gold (Illumina). Adaptor-ligated libraries were amplified and indexed via PCR.

*RNA Sequencing.* For monocytes and neutrophils up to six libraries were multiplexed per lane and sequenced at MPIMG using 100bp single end (SE) protocols following manufacturer’s instructions (V3 chemistry, HiSeq 2000, Illumina). On average each sample generated 9.18Gb of raw data (med 9.32Gb, SD 1.15Gb). For naive CD4+ T cells, libraries were prepared in the same way and sequenced at McGill university using 100bp paired-end (PE) reads, generating on average 11.74Gb of raw data (med 10.83Gb, SD 3.38Gb).

##### Data Processing

*Pre-alignment QC.* Prior to alignment reads from each RNA-seq library were initially subjected to a quality control step using FastQC (v0.10.1), where, based on duplication rates and gene coverage, outliers were identified and discarded from further analysis. Reads of monocytes, neutrophils and naive CD4+ T cells were trimmed for both PCR and sequencing adapters using Trim Galore (v0.32).

*Alignment.* Trimmed reads were aligned to the human genome using STAR (v2.4.0k) (Dobin et al., 2013). STAR default settings were used given that they were optimized for 100bp reads in human. For STAR runs, annotated splice junctions retrieved from GENCODE 15 were used to guide the alignment step.

##### Quantification of Gene Expression

To quantify and normalize gene expression, we used DESeq2 (v1.4.5) (Love et al., 2014) to obtain the read counts for each gene annotated in GENCODE 15.

##### RNA Splicing QTLs

We assessed alternative splicing using two complementary methods of quantification.

*Identification of Alternative Splicing (PSI).* To identify alternative splicing events, we used the uniquely mapped splice junction output from STAR and examined the ones that shared either the acceptor or the donor site. As described previously (Chen et al., 2014), these splicing events were compared to GENCODE 15 annotation in order to be classified as: exon-skipping, alternative 3′ splice site, or alternative 5′ splice site. Alternative splice junctions that could not be matched to any annotated splice junction were defined as ‘unannotated’. PSIs, the ratio of alternatively spliced junctions, were computed for all the alternative splicing events. We then tested association for SNPs within a 1 Mb region surrounding the PSI event, using comparable approaches to the eQTL analysis.

*Quantification of Splicing Isoform Abundance (ISO).* Splicing isoform abundance was estimated using Cufflinks (v2.2.1.) (Trapnell et al., 2010), selecting GENCODE 15 as guide annotation, without de novo transcript assembly. Cufflinks was run on RNA-Seq BAM files for monocytes, neutrophils and naive CD4+ T cells. Abundance was reported in FPKM (expected fragments per kilobase of transcript per million fragments sequenced). For ISO sQTL mapping, we employed the sQTLseekeR R package (v2.0) (Monlong et al., 2014). sQTLseekeR provides an appropriate method to identify SNPs associated with the variation in the relative abundance of a gene’s transcript isoforms, or transcript ratios, which configure a multivariate phenotype. We considered only GENCODE 15 protein coding genes that expressed at least two isoforms (thresholds for gene and transcript expression were set at 1 FPKM and 0.1 FPKM, respectively) and with a minimum splicing variability across samples. Since we were looking for cis-effects on splicing, we focused on SNPs within the gene body ± 5Kb, and separately for each cell type. To avoid testing uninformative variants, only biallelic SNPs creating at least two genotypes, each of which present in at least 5 individuals, were considered. In total, 9,485 genes and 1,462,663 SNPs were tested for association and FDR was used to correct for multiple testing. For a given transcript ratio QTL, we identified the two transcripts of the target gene that changed the most between genotypes and exhibited a symmetric behavior. Then, we employed AStalavista 3.2 software (Foissac and Sammeth, 2007) to compare their exonic structure, and determined the proportion of transcript ratio QTLs that were associated to each type of splicing event. Finally, the effect size of the identified transcript ratio QTLs was estimated as the maximum difference (MD) in relative expression between genotype groups, e.g., if MD = 0.25, there is one transcript whose relative expression shifted by 25% between two genotype groups.

##### DNA Methylation

*Sample Preparation.* Purified cells were pelleted, snap frozen and stored at −80°C prior to pre-processing at UCAM (monocyte and neutrophil) or shipping to McGill (naive CD4+ T cells).

*DNA Extraction.*

UCAM–DNA for monocyte and neutrophil samples were processed at UCAM. Cells were lysed using guanidine hydrochloride, sodium acetate and protease lysis buffer. DNA was extracted using chloroform and precipitated in ethanol prior to washing and resuspension in ultra-pure water. DNA was quantified (Qubit, Invitrogen) and shipped to University College London (UCL) for processing.

McGill–DNA was extracted from cell pellets of purified naive CD4+ T cells at McGill University using a protocol modified from the DNeasy Blood and Tissue Handbook (QIAGEN). Briefly, cell pellets were lysed using proteinase K, RNase A, and Buffer AL lysis buffer, followed by precipitation in ethanol. The DNA was purified through four successive steps with wash buffers in the DNeasy Mini spin columns, and finally eluted using a DNeasy membrane.

*Data Generation.*

UCL–500ng of DNA for each monocyte and neutrophil sample was randomly dispensed onto a 96-well plate to reduce batch effects. Samples were bisulfite-converted using an EZ-96 DNA Methylation MagPrep Kit (Zymo Research) following the manufacturer’s instructions with optimized incubation conditions (i.e., 16 cycles of 95°C for 30 s, 50°C for 60 min; followed by 4°C until further processing). Purified bisulfite-treated DNA was eluted in 15 μL of M-Elution Buffer (Zymo Research).

McGill–DNA samples were bisulfite-converted using the EZ DNA Methylation Kit (Zymo Research) according to manufacturer’s instructions.

At both institutes, DNA methylation levels were measured using Infinium HumanMethylation450 assays (Illumina) according to the manufacturer’s protocol.

*Data Processing.* Data files generated for all cell types were processed at UCL. All 450K array data pre-processing steps were carried out using established analytical methods incorporated in the R package minfi (Aryee et al., 2014). First, we performed background correction and dye-bias normalization using NOOB (Triche et al., 2013), followed by normalization between Infinium probe types with SWAN (Maksimovic et al., 2012). Next, we filtered out probes based on the following criteria: (i) median detection P value ≥ 0.01 in one or more samples; (ii) bead count of less than three in at least 5% of samples; (iii) mapping to sex chromosomes; (iv) Ambiguous genomic locations (Nordlund et al., 2013); (v) Non-CG probes; (vi) Probes containing SNPs (MAF ≥ 0.05) within 2bp of the probed CG. Finally, we adjusted for batch effects using an empirical Bayesian framework (Johnson et al., 2007), as implemented in the ComBat function of the R package SVA (Leek et al., 2012). The final data matrix used for statistical analyses, after additionally removing samples without a matching WGS sample, comprised DNA methylation M-values across 440,905 CpG sites and 525 samples, i.e., 196 monocytes, 197 neutrophils and 132 naive CD4+ T cells.

*Data Quality.* To assess the quality of the presented 450K array data and to exclude the possibility of samples mismatches, we performed a series of data quality control steps. First, we assessed the distribution of DNA methylation M-values for each cell type to identify samples of low DNA integrity (Figures S2I–S2K). Second, principal component analyses and multidimensional scaling were carried out to detect sample mismatches and outliers (Figures S2L–S2N). Third, as detailed later, we performed additional analyses to ensure that DNA methylation profiles were correctly matched to the other datasets obtained from the same donors.

##### ChIP-Sequencing

*Sample Preparation.* Purified cells were fixed with 1% formaldehyde (Sigma) at a concentration of approximately 10 million cells/ml. Fixed cell preparations were washed and either stored re-suspended in PBS at 4°C for monocytes and neutrophils, or pelleted and stored at −80°C for naive CD4+ T cells at UCAM before shipping to the processing institutes (monocytes and neutrophils to WTSI/Nijmegen Centre for Molecular Life Sciences (NCMLS), naive CD4+ T cells to McGill University).

*Chromatin Immunoprecipitation.* Chip-seq was performed at the three different institutes using different protocols (as described below and Figure S1). Naive CD4+ T cells were processed at McGill University while monocytes and neutrophils were processed at WTSI and NCMLS. For monocytes H3K27ac/H3K4me1 data, samples 1-49/1-48 are from NCMLS and 50-162/49-172 from WTSI. For neutrophil H3K27ac/H3K4me1 data, samples 1-48/1-47 are from NCMLS and 49-174/48-173 from WTSI. In these experiments antibodies from identical batches (H3K4me1, C15410194; H3K27ac, C15410196) were obtained from Diagenode (Liege, Belgium).

NCMLS–Sonication was performed using a Diagenode Bioruptor UCD-300 for 3x 10 min (30 s on; 30 s off). 67μl of chromatin (1 million cells) was incubated with 229μl dilution buffer, 3μl protease inhibitor cocktail and 1μg of H3K27ac or 0.5μg of H3K4me1 antibody and incubated overnight at 4°C with rotation. Protein A/G magnetic beads were washed in dilution buffer with 0.15% SDS and 0.1% BSA, added to the chromatin/antibody mix and rotated for 60 min at 4°C. Beads were washed with 400μl buffer for 5 min at 4°C with five rounds of washes. After washing chromatin was eluted using elution buffer for 20 min. Supernatant was collected, 8μl 5M NaCl, 3μl proteinase K were added and samples were incubated for 4 hr at 65°C.Finally samples were purified using QIAGEN; Qiaquick MinElute PCR purification Kit and eluted in 20μl EB. Illumina library preparation was performed using the Kapa Hyper Prep Kit. For end repair and A-tailing double stranded DNA was incubated with end repair and A-tailing buffer and enzyme and incubated first for 30 min at 20°C and then for 30 min at 65°C. Subsequently adapters were ligated by adding 30μl ligation buffer, 10 Kapa l DNA ligase, 5μl diluted adaptor in a total volume of 110μl and incubated for 15 min at 15°C.Post-ligation cleanup was performed using Agencourt AMPure XP reagent and products were eluted in 20μl elution buffer. Libraries were amplified by adding 25μl 2x KAPA HiFi Hotstart ReadyMix and 5μl 10x Library Amplification Primer Mix and PCR, 10 cycles. Samples were purified using the QIAquick MinElute PCR purification kit and 300bp fragments selected using E-gel. Correct size selection was confirmed by BioAnalyzer analysis.

WTSI–Sonication protocols were performed in a Diagenode PicoRuptor for 8 cycles of 30 s on, 30 s off in a 4°C water cooler. Samples were checked for sonication efficiency using the criteria of 150-500bp, by Agilent DNA bioanalyzer. ChIP-seq was carried out as previously described (Aldridge et al., 2013) all liquid handling steps were performed on an Agilent Bravo NGS. Protein A Dynabeads (Invitrogen) were coupled with 2.5μg of antibody. Sonicated lysate (3-5 million cells) was then added to the bead/antibody mix and incubated at 4°C overnight. ChIP-DNA bound beads were washed for ten repetitions in cold RIPA solution. Elution of DNA from beads at 65°C for five hours to reverse the cross linking process. 2μl RNase was added to ChIP-DNA and incubated at 37°C for 30 min, followed by 2μl of Proteinase K treatment at 55°C for 1 hr. 1:1.8 ratio of Ampure beads (Beckman Coulter, A63881) were added to the DNA followed by two cold 70% ethanol washes. ChIP-DNA was eluted in 50μl elution buffer. Illumina sequencing libraries were prepared on a Beckman Fx liquid handling system. End-repair, A-tailing and paired-end adaptor ligation were performed using NEBnext reagents from New England Biolabs, with purification using a 1:1 ratio of AMPure XP to sample between each reaction. Amplification of ChIP-DNA was performed using Kapa HiFi mastermix (Kapa Biosystems), 18 cycles of PCR followed by a 0.7:1 Ampure XP clean-up.

McGill–Sonication of nuclei was performed on a BioRuptor UCD-300 for 90 cycles, 10 s on 20 s off, centrifuged every 15 cycles, chilled by 4°C water cooler. Samples were checked for sonication efficiency using the criteria of 150-500bp by gel electrophoresis. ChIP reaction was performed on a Diagenode SX-8G IP-Star Compact using Diagenode automated Ideal Kit. 25μl Protein A beads were washed and then incubated with 3-6μg of antibody and 2-4 million cells of sonicated cell lysate combined with protease inhibitors for 10 hr, followed by 20 min wash cycle with provided wash buffers. Reverse cross linking took place on a heat block at 65°C for 4 hr. ChIP samples were then treated with 2μl RNase Cocktail at 65°C for 30 min followed by 2μl Proteinase K at 65°C for 30 min. Samples were then purified with QIAGEN MiniElute PCR purification kit as per manufacturers’ protocol. Library preparation was carried out using Kapa HTP Illumina library preparation reagents. Briefly, 25μl of ChIP sample was incubated with 20μl end repair mix at 20°C for 30 min followed by Ampure XP bead purification. A tailing; bead bound sample was incubated with 50μl buffer enzyme mix for 30°C 30 min, followed by PEG/NaCl purification. Adaptor ligation, further Ampure purification and library preparation was completed by 14 cycles of PCR amplification. Size selection was performed using a Sage Pippin prep system and set to collect 200-400bp fragments, targeting a 300bp peak fragment size and final libraries were purified with QIAGEN GeneRead Size Selection kit.

*Data Processing and Peak Calling.* ChIP libraries were sequenced using Illumina HiSeq 2000 at 50bp SE reads in WTSI, 100bp SE in McGill and 43bp SE in NCMLS. Sequenced reads were aligned to a gender-matched reference genome (Blueprint GRCh37) using BWA (*bwa aln –q 15*). Duplicate reads were marked using Picard MarkDuplicates. Reads with mapping quality less than 15 were removed (SAMtools). The fragment size L for each aligned bam was estimated using PhantomPeakQualTools vr18, which uses cross correlation of binned read counts between forward and reverse strands. To identify highly enriched genomic regions, we used MACS2 (v2.0.10.20131216, standard options) (Zhang et al., 2008) for peak calling with the estimated fragment size from PhantomPeakQualTools (*–shiftsize = half fragment size*), and with narrow and broad flags set for H3K27ac and H3K4me1 respectively. Furthermore, ChiP input was created from merging 3-12 samples, where we randomly obtained equal number of reads from each experiment. Significant peaks were selected to be at 1% FDR or less. ChIP inputs were as follows.

• Neutrophils Female (NCMLS): S001GVH2, S000X1H1, S002KJH1;
• Neutrophils Male (NCMLS): S00294H1, S001NHH1, S001C2H1;
• Neutrophils Female (WTSI): NS1140 (pool of S00W29H1, S00WP0H1, S00FK4H1) and NS1163 (pool of S00T4H1, S00NXKH1, S00PBJH1);
• Neutrophils Male (WTSI): NS1141 (pool of S00JT7H2, S00HVBH1, S00M0GH1) and NS1164 (pool of S00RMQH3, S00RD7H2, S00NRWH1);
• Monocyte Female (NCMLS): S002KJH4, S000X1H3, S001GVH4;
• Monocyte Male (NCMLS): S00294H2, S001NHH3, S001C2H3;
• T cell Female (McGill): S00DKCH4, S00G7QH2, S00GSLH2, S00GWDH2, S00JYYH2;
• T cell Male (McGill): S0021KH3, S002EVH2, S00382H2, S0064ZH2, S00D9YH1, S00DQ0H2, S00E9UH2, S00GBIH2, S00GECH2, S00HVBH2, S00JT7H1, S00KEXH3.

The majority of the neutrophil samples were immunoprecipitated at WTSI but sequenced independently at WTSI and NCMLS. For these specific samples only, we aligned each raw fastq file from the different sequencing centers to the reference genome and merge aligned bams to create only one bam for each neutrophil sample. For MACS2 peak calling of these merged samples, we used WTSI ChIP input as these samples were all immunoprecipitated at WTSI. For the case of 55 T cell H3K4me1 donors, we merged the aligned bams of duplicates of same donors in order to gain signal amplifications, as one bam alone for these donors has poor amplification.

For a complete overview of data production, refer to Figure S1.

*Data Quality.* We removed ChIP samples that had a fraction of reads in peaks (FriP) score < 0.01, relative strand correlation (RSC) < 0.8 and normalized strand correlation (NSC) < 1.05. FriP was calculated using the reference peak set that is generated as described in the next section. We identified highly successful ChIP as those with FriP > 0.01 and RSC > 0.8 and NSC > 1.05. Otherwise, we used genome browser tracks to confirm visually a good ChiP and include it in the final dataset. Figure S4 shows quality control metrics and corresponding principal components, showing no batch effects after PEER correction using K = 10 factors.

*Normalized Read Count in the Reference Peak Set.* For each histone modification marker, we generated one reference peak set for all cell types to provide an unbiased cross cell comparison of peak-based counts. For each marker, we took the union of significant peaks (1% FDR) across all donors and across all three cell types, merged overlapping regions (BEDOPS–merge, v2.4.14) and removed peaks found within ENCODE blacklisted regions. This process created one reference peak set per histone modification marker. Note that the merging process will introduce very wide peaks (≥100 KB) but they are at a very low proportion of less than 1% and 5% for H3K27ac and H3K4me1 respectively. The reference peak set will be filtered further for read counts as described below.

Next we generated quantification signal of ChiPseq for each donor. Here, we only considered read counts under the peaks, as the regions outside peaks are more likely to be noise or background signal than true ChIP enrichment. For each donor, we generated a vector of log2 reads per million (log2RPM) per peak in the reference peak set by counting the number of overlapping reads under the peaks (BEDOPS bedmap –count) and normalized the counts with the total number of reads in the library.

Note that by using only one reference peak set for all three cell types, there will be peaks where there is no signal in one cell type but quite high in another. Hence for the QTL association analysis carried out per cell type and any downstream cell-specific analyses, we further filtered the reference peak set to only consider peaks with log2RPM > 0 in at least 50% of the donors in a given cell type, corrected for 10 PEER factors and applied quantile normalization across donors.

##### Additional Quality Control to Estimate Cross-Center and Cross-Sample Identity

*Batch Correction.* Within the study, sequencing data were generated from difference sequencing centers (Figure S1). We performed the following steps to correct possible batch effects.

For RNA-sequencing and gene-level quantification, we first quantified gene expression by read count for single end RNA-seq samples, and fragment (pair) count for paired-end RNA-seq using DESeq2. The sequencing depth of different samples was then corrected by using library size factor from DESeq2. We used 15 cross-over samples to assess the impact of the different sequencing protocol, and specifically how the quantifications of single end and paired end samples correlated from the same donor in two different centers. Using PCA analysis, we observed that the cross-over samples deviated from the main clusters before ComBat, which was corrected and these samples clustered within the corresponding cell types after ComBat (Figure S3A). In Table S2 and Figure S3B, we assessed correlation in gene expression for the 15 crossover samples at different stages of the analysis (raw data, before batch effect correction using ComBat, after batch correction and finally after PEER correction). We observed a high correlation coefficient (mean 0.85) at the level of raw data. The ComBat further corrected the sequencing center effect and improved the correlation coefficient (mean 0.96), suggesting that the quantifications of single and paired-end RNA-seq were highly comparably. We observed that lowly expressed genes tended to be less well correlated. Therefore, in the QTL analysis, we further required that a gene to have more than 10 read count in 50% of the samples. Furthermore, we applied PEER to infer and correct for 10 hidden factors.

For PSI quantification, we found the crossover samples (see later) to display the greatest differences for low quantification values (PSI from 0 to 0.1), with low overall correlations in pairwise comparisons (mean 0.556). We therefore requested PSI quantification to be 0.1 in 50% of samples. After removing the high noise in the low PSI, the correlations were improved to 0.920 (Table S2). PSI values had bimodal distribution and we standardized and applied PEER to infer and correct for 10 hidden factors (Figure S3C).

For 450K methylation arrays, we used identical protocols across the two production centers (UCL and McGill). We again compared the correlation between nine crossover samples between the two centers. The average correlation coefficient of normalized beta value before correlation is 0.959. After applying ComBat correlation using the sequencing center as a covariate, the average correlation coefficient increased to 0.994 (Table S2). After ComBat, we further examined the M-value distribution, PCA analysis and found that the cross-over samples fitted into their corresponding distribution and PCA in three cell types (Figures S2I–S2N).

For Chip-sequencing data, we generated one reference peak set per histone modification mark and obtained the log2 RPM (reads per million of sequencing depth) under each peak. In Table S2 and Figure S4E, we show high Pearson correlation (0.86 - 0.97) for 2-3 cross over replicates in two different ChIP centers. Furthermore, we show in Figure S4E that the samples cluster correctly into their correct cell types. To remove the ChIP center bias that is present in monocytes and neutrophils only, we carried out PEER-correction for 10 hidden factors on the log2 RPM peak signals and show in Figures S4G–S4J uniform density profiles across all samples and PCA plots devoid of ChIP center effect. The PEER-corrected signal is used for the QTL association analysis of each cell type, where we further filtered the peaks to only consider peaks with log2 RPM ≥ 1.

*Cross-Center Data Validation.* In addition to the samples processed in the above RNA-seq and DNA methylation experiments, three samples of each cell type per sample batch were sent to the reciprocal institute that did not process that particular cell-type specific sample set, in order to account for institute-specific experimental variation. For RNA-seq naive CD4+ T cells were sent to MPIMG, monocytes and neutrophils to McGill University; for DNA methylation naive CD4+ T cells were sent to UCL, monocytes and neutrophils to McGill University.

For RNA-seq, sequencing was done in different centers using either single-end or paired-end protocols. To evaluate whether we could compare the RNA-seq data cross centers and library protocols, we quantified the cross-over samples and adjusted for batch effects using sequencing centers and library protocols as covariates in ComBat (Chen et al., 2011). We subsequently performed principal component and multidimensional scaling analysis on the adjusted data. As shown in Figure S3, the crossover samples (indicated in a darker color) clustered with the main cell-type specific sample set, demonstrating successful correction of any confounding by institute-specific experimental variation.

For DNA methylation, we adjusted for batch effects as described above, and subsequently performed principal component analyses and multidimensional scaling on the adjusted data. The crossover samples (indicated in a darker color) clustered with the main cell-type specific sample set, demonstrating successful correction of any confounding institute-specific experimental variation (Figures S2M and S2N).

*Confirmation of Sample Identity across Datasets.* Identity matching for each sample and for each analysis was performed by extracting genotypes from RNA-seq and ChIP-seq and comparing them to SNPs from the WGS data. The first stage of verifying the sample identity concordance between the RNA-seq/Chip-seq and WGS data involved pre-processing the BAM files for one autosomal chromosome (chr1) to remove PCR duplicates and reads with mapping quality score < 10. The variants were then called from the resulting BAM file using *mpileup* from the SAMtools package (Li, 2011). The variants with QUAL < 20, DP < 5 and GQ < 5 were filtered out. Then, we compared genotypes of the filtered variants with genotypes generated from WGS and imputation. The genotypes generated were considered to be from the same sample if the concordance rate was greater than 90%.

For DNA methylation, first we estimated sex and age of all samples based on raw DNA methylation values using the getSex function in minfi (Aryee et al., 2014) and the DNA Methylation Age Calculator as described by Horvath (Horvath, 2013), respectively. We then correlated the estimated information from the experimental data to the information collected from each donor, to confirm sample identity. Second, for the 65 SNPs from methylation probes (the internal controls) on the chip, we derived the genotypes from raw beta values: raw beta value < 0.3 for homozygous AA or TT genotype call, beta value > 0.7 for homozygous CC or GG genotype, the remainder were classified as heterozygous genotypes. We then checked sample identity by comparing these inferred genotypes to their genotype from WGS.

Finally, we used PCA and unsupervised clustering methods to verify that each RNA-seq, Chip-seq and methylation sample matched its predicted cell type of origin. All sample identities and types were confirmed prior to uploading the data files to the EGA.

##### Summary of Dataset

The dataset used for the analysis consists of 2,205 samples across all assays from 197 unique donors (Table S1). This breaks down as follow; WGS 197 samples; RNA-seq 194/192/171 monocytes/neutrophils/CD4+ T cells respectively; DNA methylation 196/197/133 monocytes/neutrophils/CD4+ T cells respectively; ChIP-seq H3K4me1 172/173/104 monocytes/neutrophils/CD4+ T cells respectively; ChIP-seq H3K27ac 162/174/142 monocytes/neutrophils/CD4+ T cells respectively.

### Quantification and Statistical Analysis

#### Statistical Analyses

##### Variance Component Modeling of Gene Expression

To investigate the contributions to gene expression variability from different proximal molecular features we considered different variance component models fit using LIMIX (Casale et al., 2015, Lippert et al., 2014).

For all variance component analyses, we considered only individuals for which data for all molecular layers (gene expression, DNA, methylation, H3H4me1 and H3H27ac) were available. For T cells we excluded the ChIP-seq data, as the matching would have reduced the dataset size to less than 100 samples. The resulting matched dataset consisted of gene expression profiles for 16,549, 14,986 and 17,802 genes in 158, 165 and 125 individuals respectively in monocytes, neutrophils and CD4+ T cells.

*Independent Variance Component Models for Epigenetic and Genetic Effects on Gene Expression.*

Accounting for Confounding–When correlating transcriptional and epigenetic variation, there is a concern that sample processing effects and other sources of heterogeneity may be shared between gene expression levels and epigenetic profiles, thereby introducing spurious correlations. To mitigate such confounding factors, we applied PEER and used residual profiles for gene expression levels, DNA methylation and histone modification marks (PEER (Stegle et al., 2012) was fit using 10 factors as described above). PEER residuals were quantile-normalized to a unit variance Gaussian distribution. To further reduce the risk of confounding correlations we additionally considered a random effect term in our model that accounts for transcriptome heterogeneity not captured by PEER. Specifically, the sample covariance of this expression heterogeneity term was estimated as $K_{h}=\left(1/G\right)ZZ^{T}$, where Z is N×G matrix of gene-expression levels for N individuals and all G genes (after quantile-normalization of the distribution of PEER residuals for each gene to a unit variance Gaussian distribution).

Without Correction for Local Genetic Effects–For each gene, we considered the model

(1) $$y=N\left(1\mu ,\sigma_{l}^{2}K_{l}+\sigma_{g}^{2}K_{g}+\sigma_{h}^{2}K_{h}+\sigma_{e}^{2}I\right),$$

where y denotes the gene-expression profile across individuals, 1μ an offset term, $K_{l}$ is a local relatedness matrix built using all features from either one of the four molecular layers (genetic, methylation, H3K4me1 or H3K27ac data) that are within 1Mb from the gene body, $K_{g}$ denotes the realized relatedness matrix (Lee et al., 2010), $K_{h}$ is the expression heterogeneity term and $\sigma_{e}^{2}I$ is the noise term. Specifically, the local relatedness matrix for each feature type was estimated as linear kernel from all *cis* features of the considered type (after standardization).

The variance parameters $\sigma_{l}^{2}$, $\sigma_{g}^{2}$, $\sigma_{h}^{2}$ and $\sigma_{e}^{2}$ were fitted using restricted maximum likelihood, independently for each of 16,549, 14,985 and 17,082 genes in monocytes, neutrophils and naive CD4+ T cells. The log restricted marginal likelihood was optimized using a gradient-based optimization algorithm (BFGS) (Morales and Nocedal, 2011). The proportion of variance explained by individual components was then estimated analogous to the approach taken in classical (narrow sense) heritability analysis (Yang et al., 2011):

$$h=\frac{\sigma_{l}^{2}}{\sigma_{l}^{2}+\sigma_{g}^{2}+\sigma_{h}^{2}+\sigma_{e}^{2}}$$

When comparing variance component estimates of the model in (1) with a model that does not account for expression heterogeneity, we found that accounting for expression heterogeneity yielded substantially lower epigenome variance estimates, whereas the genetic variance estimates were unaffected (Figure S5J). Consequently, we considered a model that accounts for expression heterogeneity in all subsequent analyses. We also considered alternative window sizes (100kb and 1Mb), finding that the results were most robust and that the overall variance was slightly increased when using 1MB window sizes (Figure S5K).

Accounting for Local Genetic Effects–To account for *cis* common genetic variation, we first corrected epigenetic features for local genetic effects. To do so we fitted a separate variance component model for each individual epigenetic feature, using a local relatedness matrix based on all SNPs within 100Kb from the epigenetic mark. The effect from local genetic variants was estimated using the best linear unbiased predictor and the residuals of this model were then used as an estimate of the non-genetic component of the epigenetic marks (G-corrected marks). Additionally, we introduced a random effect in the model to account for genetic effects on gene expression from variants within 1Mb from the gene body. Specifically, for each gene we considered the model

(2) $$y=N\left(1\mu ,\sigma_{l}^{2}K_{l}+\sigma_{\text{geno}}^{2}K_{\text{geno}}+\sigma_{g}^{2}K_{g}+\sigma_{h}^{2}K_{h}+\sigma_{e}^{2}I\right),$$

Here, $K_{\text{geno}}$ is a local realized relatedness matrix built considering all genetic variants in 1Mb from the gene-body and $K_{l}$ is a local relatedness matrix built considering all features from either one of the three epigenetic layers (methylation, H3K4me1 or H3K27ac data) that are within 1Mb from the gene body. This model was used to estimate the proportion of variance explained by methylation, H3K4me1 and H3K27ac data while accounting for underlying genetic effects.

The cumulative distribution of the proportion of variance explained by local genetics (using model (1)) and each of the three epigenetic layers either accounting (model (2)) or not accounting (model (1)) for local genetic effects is shown in Figure 2B for monocytes, Figure S5A for neutrophils and Figure S5B for T cells.

*Joint Variance Component Model.*

For each gene, we also considered variance component estimates obtained from a joint model across all four molecular layers (genetics, methylation, H3K4me1 or H3K27ac)

$$y=N\left(1\mu ,\sigma_{\text{geno}}^{2}K_{\text{geno}}+\sigma_{\text{meth}}^{2}K_{\text{meth}}+\sigma_{\text{K}4\text{me}1}^{2}K_{\text{K}4\text{me}1}+\sigma_{\text{K}27\text{ac}}^{2}K_{\text{K}27\text{ac}}+\sigma_{g}^{2}K_{g}+\sigma_{h}^{2}K_{h}+\sigma_{e}^{2}I\right).$$

Here, the local relatedness matrix for each layer were computed considering all G-corrected epigenetic marks within 1Mb from the gene body relative to the specific layer. Epigenetic variance estimates were either considered for individual layers or by aggregating using the sum of the variance components across the three epigenetic layers. The distribution of the total epigenetic contribution to variance is shown in Figure 2C (y axis) for monocytes, Figure S5G (y axis) for neutrophils and Figure S5H (y axis) for T cells.

##### Testing for Variance Components

To test for *cis* genetic contributions (within 1Mb from the gene-body), we considered the model

$$y=N\left(1\mu ,\sigma_{\text{geno}}^{2}K_{\text{geno}}+\sigma_{g}^{2}K_{g}+\sigma_{h}^{2}K_{h}+\sigma_{e}^{2}I\right),$$

and tested for $\sigma_{\text{geno}}^{2}>0$. To test for *cis* contributions from methylation, H3K4me1 peaks and H3K27ac peaks that are independent from *cis* common genetic variation, we used the model in (2), where the local relatedness matrix $K_{l}$ was built considering either methylation, H3K4me1 or H3K27ac features (again within 1Mb from the gene body) after correction for local genetic effects, and tested for $\sigma_{l}^{2}>0$. We considered log likelihood ratio (LLR) as test statistics and obtained p values using permutations, similar to the approach in (Casale et al., 2015, Lippert et al., 2014). Specifically, we considered 30 permutations for each test and gene and combined null LLRs across all genes. This resulted in a total of ∼600,000 permutation LLRs for each epigenetic layer and cell type, which we used to estimate empirical P values (minimum pv ≈1.7 ^∗^ 10^−6^). Empirical P values were corrected for multiple testing using the Benjamini-Hochberg procedure. Significant associations with gene expression levels were reported at an overall FDR of 5%. Results from the variance component tests are shown in Figures 2C–2E for monocytes, Figure S5G for neutrophils and Figure S5H for T cells.

##### Epigenome-wide Association Analysis of Gene Expression

To differentiate epigenetic associations with gene expression that are due to underlying local genetic variation from associations that are independent of genetic effects, we also carried out classical single-feature association tests, with and without adjusting for genetic factors in the model. For both models, we considered associations between gene expression level and all epigenetic features that are in 1Mb from the gene body.

*Uncorrected EWAS Model.* To test for association between gene expression and epigenetic features within 1Mb from the gene body (including methylation and histone modification) we consider the following linear mixed model:

$$y=N\left(1\mu +e\beta ,\sigma_{g}^{2}K_{g}+\sigma_{h}^{2}K_{h}+\sigma_{e}^{2}I\right)$$

Here, **y** denotes the gene-expression profile across individuals for gene g, **1**μ an offset term, ***e*** is the specific epigenetic feature of interest, $K_{g}$ denotes the realized relatedness matrix (Lee et al., 2010), $K_{h}$ is the expression heterogeneity term and $\sigma_{e}^{2}I$ explains residual variance. All epigenetic features and gene-expression levels were quantile-normalized to unit variance Gaussian distribution prior to testing for associations.

*G-Corrected Model.* Proceeding as in the variance component analysis, we considered the model:

$$y=N\left(1\mu +e^{\prime }\beta ,\sigma_{\text{geno}}^{2}K_{\text{geno}}+\sigma_{g}^{2}K_{g}+\sigma_{h}^{2}K_{h}+\sigma_{e}^{2}I\right),$$

where ***e***’ is the G-corrected genetic feature being tested and $K_{\text{geno}}$ is a local realized relatedness matrix built considering all variants in 1Mb from the gene-body. G-corrected epigenetic features were also quantile-normalized to a normal distribution prior to association testing.

Association testing was performed using LIMIX (Casale et al., 2015, Lippert et al., 2014). For both models, variance components were estimated under the null model and only the total variance was updated during the association testing (Kang et al., 2008b). For multiple hypothesis correction, we performed a two-step procedure (Battle et al., 2014): we first obtained a gene-level P value as the minimum nominal P value (Bonferroni corrected to account for multiple testing across *cis* features) and then used the Q-value procedure (Storey and Tibshirani, 2003) to correct for multiple testing across genes. We called genes with significant epigenetic association at FDR < 5%.

##### QTL-Mapping

*Gene, Methylation, Histone Modification QTL Mapping. Cis*-acting QTL mapping was done using the LIMIX package. We considered genetic variants mapping to within 1 Mb (on each side) of each tested feature, and tested their association with gene expression, splicing (percent splice in, PSI), methylation levels and histone modification peaks (H3K27ac and H3K4me1).

Linear regression models were fit between the genotypes and trait quantification, also including a random effect term accounting for polygenic signal and sample relatedness (as in the variance component models above we used the realized relatedness matrix to capture sample relatedness). Analogously to the variance decomposition analysis, we considered quantile-normalized PEER residuals for this analysis. From the linear regression, we obtained the effect size and p value for each tested association.

To correct for multiple hypothesis testing, we performed a two-step procedure (LRVM) (Battle et al., 2014): first, we corrected for multiple testing across variants for each molecular outcome using Bonferroni correction and, second, we adjusted the obtained p values for multiple-testing across phenotypes within each layer using the Q-value procedure (Storey and Tibshirani, 2003), considered QTLs at a significance threshold of 5% FDR.

##### Allele-Specific Expression (ASE) Mapping

To assess allele-specific expression (ASE) mapping in a similar manner to QTL mapping (above), the aligned RNA-seq reads (to hg19 reference genome) were divided into separate BAM files based on forward and reverse orientations. Read from only the forward strand were used in analysis of transcripts in forward orientation, and reads from reverse strand were used for analysis of reverse orientation transcripts. PCR duplicates were filtered out from all subsequent analysis.

In total 672,115,720, 623,962,195 and 496,318,001 filtered reads with allelic information were available in neutrophils (n = 196), monocytes (n = 194) and naive CD4+ T cells (n = 169), respectively. Allelic expression from RNA-seq reads at all heterozygous SNPs was counted with customized python code using the Pysam package. We adjusted for reference bias caused by genome alignment by using only heterozygous SNPs with reads in both alleles in the sample. This requirement that both alleles at heterozygous sites were observed reduced the overall number of informative reads by 8.5%. On average, there were 138K heterozygous expressed sites per individual with both alleles observed in RNA-seq and with a mean count of ∼20 reads per site per sample. To further reduce the reference allele bias, we re-mapped the reads to filter out potential problematic reads by using the WASP (van de Geijn et al., 2015), which almost completely removed the reference allele bias (final bias 50.1%). This step also reduced informative sequence by 15% on average.

After WASP correction, we performed two types of allele-specific testing: a) linear regression and b) CHT.

*Linear AS Test.* The sum of allelic counts of each haplotype in the gene region was used to calculate its ASE ratio. The samples used in ASE mapping for each gene required at least two informative SNPs (heterozygous genotype with reads in both alleles) with ≥ 10 reads in each SNP in the gene region. Given that ASE mapping can be sensitive to outlier effects when few heterozygotes are available for analyses, we applied a cutoff of MAF ≥ 0.05 for the tested SNP and more than 5 samples with ASE in the gene region. We performed ASE-mapping by using those allele ratios in regression testing for local SNP association (250kb flanking each side (TSS/TES) of the transcript), as was previously described in a linear regression model (Ge et al., 2009). In aggregate, a total of 14,962 gene loci were tested. The gene region definition is the same as the one used in our earlier eQTL analysis. We also carried out the conditional (secondary) AS mapping by performing a linear AS test using gene allele ratios from samples with homozygous genotype in lead SNP in the primary mapping. All genes with the most significant p value at 5% FDR were tested.

Combined Haplotype Test (CHT). The analyses were carried out using the combined haplotype test in WASP (van de Geijn et al., 2015), as per author’s instructions. A minimum of 20 samples with allele-specific data in the gene region was required for the CHT testing. The first 4 principal components, generated from PCA, were also used as covariates in the CHT. In total, 19,283 gene loci were tested.

In the linear AS test, one sample contributes to a single data point (average ratio) in the final statistical testing while in the CHT, each heterozygous SNP site with allele reads is one data point. Therefore, CHT allows us to test more features.

##### Allele-Specific Histone Mark (ASH) Mapping

The methods and steps are very similar to those described for ASE (above), with the exception that sequence orientation is not required. After filtering for duplicates, we obtained a total of 154,674,565, 143,357,603 and 373,066,694 sequence reads with allelic information for H3K4me1 and 122,631,444, 105,172,159 and 203,402,978 sequence reads with allelic information for H3K27ac in neutrophils, monocytes and naive CD4+ T cells, respectively. We noticed that WASP correction removed less than 5% of informative sequence reads in ChIP-seq, which is significantly lower than the 15% observed in the RNA-Seq dataset. Similar mapping methods to ASE (described above) were applied to histone mark reads. A total of 36,729 and 38,546 peak regions were analyzed with the linear AS test for H3K27ac and H3K4me1 histone marks separately. With the CHT method, 70,894 and 45,867 peaks were tested for H3K27ac and H3K4me1, respectively.

In the end, we obtained 18 sets of AS mapping data (6 sets from ASE and 12 sets from ASH). We performed false discovery rate (FDR) estimation from the p values for each dataset, using the qvalue package in R. FDR (or qvalue) were used for later comparisons between datasets.

In order to assess the regulatory SNPs shared between genes and histone peaks, we used two different approaches. The first approach is based on LD information between the two lead SNPs from ASE and ASH mapping data, respectively. We define the gene and peak pair sharing the same regulatory element if two the lead SNPs are in LD (r^2^ ≥ 0.8). The LD r^2^ values were calculated from our phased SNP genotype dataset (197 samples).

The second approach is based on correlation test between the allele ratios of the gene and histone mark. We extracted allele ratios from both sets and from shared samples for all pairs of genes and histone peak pairs at a distance of less than 1Mb. We required a minimum of 25 samples with 2 informative SNPs and a minimum of 10 reads in both RNASeq and ChIPseq data. On average, 4% of tested pairs have correlation |r| ≥ 0.3 or p value < 0.05.

Overall, 60% of pairs of genes and histone peaks in LD were confirmed by correlation of allele ratios in the linear AS test set. However, only 30% of pairs were confirmed in the CHT set and 15% of pairs in the QTL set. Since the average ratios were used in both linear AS mapping and allele ratio correlations, this set has much higher concordance rate than that in other sets as expected.

The phased allele ratio was also used to verify ASE mapping results. Allele ratio values that were shared in all three cell types from 166 samples were extracted. To obtain more reliable results, we required a minimum of 3 SNPs in the gene region with at least 40 reads each for all genes with the most significant p value at 10% FDR. The allele ratios between lead SNP homozygous and heterozygous groups should have significant difference.

##### Annotation and Comparative Analyses

*QTL Sharing and Cell-type Specificity.* Based on the π1 statistic (Storey and Tibshirani, 2003) and the procedures in (Nica et al., 2011), QTL sharing was estimated as the proportion of true associations *π*_*1*_ among the QTLs from a first cell type in a second cell type. We employed *qvalue* to compute *π*_*1*_ as *1−π*_*0*_, where *π*_*0*_ is the estimated proportion of truly null associations. Cell type specificity was estimated as 1 minus the average of *π*_*1*_ values from one cell type in the others.

*Enrichment of ISO QTLs in Biologically Relevant Features.* We tested for ISO QTLs (FDR ≤ 5%) mapping to exons, and splice sites more often than non-ISO QTLs (FDR > 5%) with matched minor allele frequencies. We also compared the distance to the closest exon for intronic ISO QTLs and non-ISO QTLs.

*GWAS Annotation and Enrichment in QTL Overlaps.* To overlap our molecular QTLs to GWAS disease variants, we use the full summary statistics of selected seven autoimmune diseases: celiac disease [CEL] (Dubois et al., 2010), inflammatory bowel disease [IBD] (Liu et al., 2015), including Crohn’s disease [CD] (Liu et al., 2015) and ulcerative colitis [UC] (Liu et al., 2015), multiple sclerosis [MS] (Beecham et al., 2013), Type 1 diabetes [T1D] (Onengut-Gumuscu et al., 2015), and rheumatoid arthritis [RA] (Okada et al., 2014)). The associations of IBD, CD and UC in the European cohorts were used for this study. We also used Type 2 diabetes (Morris et al., 2012) as a negative control. If the lead QTL (≤5% FDR) or its LD tag (r^2^ ≥ 0.8) maps to a GWAS variant (P value ≤ 5x10^−8^), then we consider that the QTL overlaps with a GWAS signal. Here, we calculated the LD information of the QTLs based on our WGS data using plink (Purcell et al., 2007) and 500 kb window.

In order to systematically measure the statistical significance of the overlaps between GWAS disease variants and molecular QTLs, we used GARFIELD (Iotchkova et al., 2016), a novel enrichment analysis approach taking genome-wide association summary statistics to calculate odds ratios for association between annotation overlap and disease status at given GWAS significance thresholds, while testing for significance via generalized linear modeling framework accounting for linkage disequilibrium, minor allele frequency, and local gene density. Linkage disequilibrium was calculated using SNPs from the combined UK10K and 1000 genomes Phase3 European cohorts. For functional annotations, we used the genomic positions of unique significant variants (5% FDR) for each QTL type (gene expression, splicing, methylation, H3K27ac and H3K4me1) in all three cell types. We tested for enrichment variants reaching 1x10^−5^ significance threshold for selected autoimmune diseases as listed above. Multiple testing correction was further performed on the effective number of annotations used.

*Colocalization between Diseases and Molecular Trait.* We used a Bayesian colocalization method (Giambartolomei et al., 2014, Pickrell et al., 2016) to elucidate whether the observed overlap between disease and molecular trait may due to a shared genetic effect. The method calculates the posterior probability (PP), versus the null model of no association, for four alternative models: a model where a region or locus contains a single variant associated with either the molecular trait or disease (models 1,2); a model where a single causal variant affects association with both traits (model 3); or a model where two distinct associations exist (model 4). The method derives the PP of each variant in the locus being causal one under different models, and the PP of a given locus is then the integral sum of the PPs of all variants within, with all variants under equal prior probability to be causal. The prior for each model is computed to be one that maximizes the log-likelihood function (Pickrell et al., 2016). We acknowledge the limitations of the model: it assumes one causal variant in the locus; and in the case of high LD between two causal variants the model has limited power to distinguish model 4 from model 3. We also note that colocalization does not imply a causal relationship between molecular trait and diseases, but may be compatible also with the same variant having independent (‘pleiotropic’) effects on molecular traits and disease. We applied colocalization test for each of the 1,003 disease-molecular trait pairs, where the lead SNPs in both traits are in high. r^2^ ≥ 0.8. To avoid overlapping 2Mb-wide genetic loci due to features in close proximity (e.g., splicing junctions, genes, histones peaks, CpGs in islands), we tested colocalization per locus, which means that the prior model parameters were estimated using one locus instead of multiple loci and hence the priors may be overestimated.

*Integration with Blueprint ChromHMM Segmentation States.* We used the reference Blueprint chromatin segmentation states for the three cell types in this study, full methodology is described here (Carrillo de Santa Pau et al., 2016). For each cell type, the chromatin states were inferred using ChromHMM (Ernst and Kellis, 2012) and six histone modification markers: H3K4me3, H3K36me3, H3K27ac, H3K4me1, H3K27me3 and H3K9me3. The 11 chromatin states are: E1 for Transcription Low signal H3K36me3; E2 Transcription High signal H3K36me3; E3 Heterochromatin High Signal H3K9me3; E4 Low signal; E5 Repressed Polycomb High signal H3K27me3; E6 Repressed Polycomb Low signal H3K27me3; E7 Repressed Polycomb TSS High Signal H3K27me3 & H3K4me3 & H3K4me1; E8 Enhancer High Signal H3K4me1; E9 Active Enhancer High Signal H3K4me1 & H3K27Ac; E10 Active TSS High Signal H3K4me3 & H3K4me1; E11 Active TSS High Signal H3K4me3 & H3K27Ac. For each cell type, we then merged (Bedtools multiIntersectBed) the chromatin states from multiple replicates (2 monocytes, 8 neutrophils, 6 T cells) requiring that the state is present in at least 50% of the samples. Hence, we only used one reference chromatin state per cell type.

### Data and Software Availability

#### Data Resources

The full QTL summary statistics from this study can be accessed from http://blueprint-dev.bioinfo.cnio.es/WP10/qtls. The accession numbers for the alignment data reported in this paper are European Genome-phenome Archive (EGA): EGAD00001002663 (WGS), EGAD00001002671/EGAD00001002674/EGAD00001002675 (RNA), EGAD00001002670/EGAD00001002672/EGAD00001002673 (ChIP-seq) and EGAS00001001456 (450K DNA methylation). Quantification matrices, donor metadata, Chip-seq peaks and Chip-seq coverage files are available via ftp://ftp.ebi.ac.uk/pub/databases/blueprint/blueprint_Epivar/.

### Additional Resources

Chromatin immunoprecipitation protocols: http://www.blueprint-epigenome.eu/index.cfm?p=7BF8A4B6-F4FE-861A-2AD57A08D63D0B58

## Author Contributions

Conceptualization, L. Chen, T.P., O.S., N.S., and H.G.S.; Methodology, V.A., S.B., L. Chen, O.D., K.D., S.E., M.F., D.G.-M., R.G., H.L., J.H.A.M., D.S.P., A.R., D.R., N.S., T.P., M.S., A.V., L.V., S.P.W., and M.-L.Y.; Software, J.A.M., J.M.F., L. Chen, D.G.-M., R.G., V.I., K.K., A.V., and L.V.; Validation, L. Chen and K.D.; Formal Analysis, C.A.A., V.A., S.B., M.C., L. Chen, W.C., K.D., S.E., H.E., D.G.-M., B.G., E.H., V.I., K.K., T.K., A.L.M., V.P., T.P., D.S.P., D.R., T.R., X.S., N.S., L.V., K.W., Y.Y., and M.-L.Y.; Investigation, T.W.K., I.C., F.P., M.-M.S., S.-Y.W., F.O.B., S.A., K.B., F.B., S.-H.C., L. Chen, K.D., S.E., S.F., M.F., D.G.-M., R.G., E.J.-M., B.K., J.J.L., A.M., J.H.A.M., F.M., V.P., F.P.C., A.R., K.R., D.R., S.R., N.S., M.S., N.S., T.P., L.V., and S.W.; Resources, M.T.M., V.A., L. Chen, A.D., K.D., S.E., S.F., M.F., F.J., H.L., E.L., J.H.A.M., T.P., D.S.P., F.P.C., D.R., D.R., N.S., L.V., S.W., S.P.W., Y.Y., and M.-L.Y.; Data Curation, V.A., L.B., D.B., M.C., L. Chen, W.C., A.D., K.D., S.F., B.G., F.J., H.H.D.K., E.L., J.H.A.M., T.P., D.S.P., D.R., N.S., L.V., S.W., and Y.Y.; Writing – Original Draft, L. Chen, T.P., O.S., N.S., and L.V.; Writing – Review & Editing, L. Chen, L.V., A.L.M., V.P., P.C., O.S., T.P., and N.S.; Visualization, J.A.M., L. Chen, S.F., T.K., B.G., and L.V.; Supervision, S.B., G.B., L. Clarke, K.D., S.F., P.F., M.F., R.G., J.H.A.M., W.H.O., T.P., D.R., N.S., H.G.S., A.V., L.V., and M.-L.Y.; Project Administration, G.B., K.D., S.F., M.F., D.M., and N.S.; Funding Acquisition, S.E.A., S.B., G.B., E.T.D., S.F., P.F., M.F., W.H.O., T.P., N.S., and H.G.S.

## Supplemental Information

Table S1. Donor Characteristics and Per-Sample Data Quality Metrics, Related to Figure 1 Details of donor characteristics (gender, smoking status past and present and age bin), identification (ID) code and donation date along with FACS purity results by cell type and sample quality metrics for each assay. Missing data are indicated by ‘no data’, ‘-’ show samples that were removed due to assay and/or QC fails. N.A. is shown for FACS data where no samples of this cell type were used.

Table S2. Crossover Samples and Batch Correction, Related to Figure 1 Crossover sample information and correlation coefficient before and after batch correction in crossover samples of RNA-seq and 450K methylation array. In Chip-seq, as ComBat cannot be applied, we used a normalization method based on library size.

Table S3. Overview of Genes Identified as Having a Significant Epigenetic 0r Genetic Contribution in Three Cell Types, Related to Figures 2, 3, 4, 5, and 6 For each cell type, we report whether a given gene was associated as carrying a genetic or epigenetic feature in each of the following analyses: VD=Variance Decomposition, further divided in epigenetic contribution independent of genetics (at Meth, H3K27ac or H3K4me1 sites), and genetic contribution (VD_geno); gene level eQTL, allele-specific analysis (AS expression, ASE or combined haplotype test, CHT) and splicing QTL (ISO or PSI). “1” indicates a significant association, “0” lack of association and “NA” that the gene was not tested (for instance if not expressed, if not alternatively spliced, or for not meeting any other inclusion criteria in the study).

Table S4. Canonical Pathways Enrichment for Genes under Genetic and Epigenetic Control, Related to Figures 2, 3, and 4 For ‘Epigenetic’ rows show genes identified by the variance decomposition analysis as having a significant epigenetic contribution independent from cis-genetic effects. Genetic rows detail genes identified as having a significant genetic contribution in eQTL or sQTL analyses.

Table S5. Summary of Colocalization Results in the Genetic Association of Autoimmune Diseases and Molecular Traits, Related to Figure 7 List of lead QTLs (gene, PSI, DNA methylation and histone modifications) loci that colocalize a variant associated with selected autoimmune diseases [multiple sclerosis (MS), Celiac disease (CEL), rheumatoid arthritis (RA), Crohn’s disease (CD), inflammatory bowel disease (IBD), ulcerative colitis (UC), Type 1 diabetes (T1D)] and Type 2 diabetes (T2D)]. Column labels PP1 - PP4 are the posterior probability that a given locus contains a causal variant that follows a model of single association [PP1, PP2], a model of shared association of two traits [PP3] or a model with two causal variants of independent associations [PP4]. Likewise, logBF_i and pi_i are the log Bayes factor and prior probability respectively for the different models i=1,2,3,4.

Table S6. Genetically Controlled Gene-Histone Mark Peaks Validated by Orthogonal Allelic Correlation, Related to Figures 3, 5, and 6
